# Red pulp macrophages clear parasites, while marginal metallophilic and marginal zone macrophages support CD4^+^ T cell activation during *Plasmodium yoelii* infection

**DOI:** 10.3389/fimmu.2025.1607201

**Published:** 2025-07-17

**Authors:** Julia Falkenstein, Anne Ninnemann, Matthias Hose, Abdelrahman Elwy, Karl S. Lang, Kai Matuschewski, Jan Buer, Astrid M. Westendorf, Wiebke Hansen

**Affiliations:** ^1^ Institute of Medical Microbiology, University Hospital Essen, University Duisburg-Essen, Essen, Germany; ^2^ Institute of Immunology, Medical Faculty, University of Duisburg-Essen, Essen, Germany; ^3^ Institute of Biology/Faculty for Life Sciences, Molecular Parasitology, Humboldt University Berlin, Berlin, Germany

**Keywords:** splenic macrophages, *Plasmodium yoelii*, parasite clearance, T cell responses, malaria immunity

## Abstract

Malaria, caused by the parasite *Plasmodium* spp., remains the most prevalent and dangerous vector-borne infectious disease worldwide. Effective pathogen clearance during malaria hinges on the interplay between adaptive and innate immune responses, especially on T cells, B cells, antigen-presenting cells (APCs) and IFNγ response. In a previous study, we demonstrated that dendritic cell (DC) depletion resulted in impaired T cell responses. However, substantial CD4^+^ and CD8^+^ T cell activation was still detectable, suggesting that other APCs compensate for the lack of DCs. In the present study, we report an increase in splenic marginal zone macrophages (MZMΦ), and marginal metallophilic macrophages (MMMΦ) with an altered cytokine profile in DC-deficient mice upon *P. yoelii* infection. Ablation of macrophages by clodronate liposome (CL) application resulted in partially reduced T cell activation, which correlated with elevated parasitemia. To further elucidate the specific role of splenic macrophage subsets we studied *P. yoelli* infections in two transgenic C57BL/6 mouse lines. Treatment of CD169DTR mice with diphtheriatoxin (DT) efficiently depleted MMMΦ and MZMΦ, resulting in reduced IFNγ production by CD4^+^ T cells in *P. yoelii*-infected mice, though parasitemia progression was not modulated. In marked contrast, specific red pulp macrophages (RPMΦ) depletion in SpiC^flox/flox^ x vav1cre mice resulted in elevated parasitemia. In conclusion, our data provide evidence that splenic macrophages located in or at the marginal zone contribute to CD4^+^ T cell activation, and that RPMΦs are indispensable for clearing of infected red blood cells (iRBCs) during *P. yoelii* infection.

## Introduction

Malaria is caused by the *Plasmodium* parasite and is one of the most prevalent and dangerous infectious diseases globally (1). Despite extensive research, the dynamics and connectivity of innate and adaptive immune responses elicited by a first *Plasmodium* blood infection remain largely elusive. Thus, there is an urgent need to enhance the understanding of the immune response to the parasite to develop new vaccines or therapeutics. Key effector mechanisms controlling parasite replication in the early phase of infection include macrophage activation, T cell activation, B cells for antibody production and IFNγ production (2–4). T cells have to be activated by professional APCs, which initiate a cascade of downstream signaling pathways, leading to the proliferation of naïve cells and their differentiation into specialized effector cells (5–7). DCs are regarded as the most critical APCs due to their enhanced ability to activate naïve T cells (8), and CD4^+^ T cells play a central role in orchestrating immune responses against *Plasmodium* parasites (9–11). In particular, CD4^+^ T helper 1 (T_H_1) cells that secrete IFNγ and induced type 1 regulatory (Tr1) cells that produce IL-10 not to help mitigate the pathology caused by the inflammatory response and regulate progression of *Plasmodium* blood infection (12). Furthermore, CD4^+^ T cells support B cell responses, which are capable to drive macrophage activation. This dual function facilitates the elimination of iRBCs during the spleen passage, thereby contributing to infection control (13). There is evidence from studies in both humans and mice, indicating that DC function may be impaired during blood-stage infections, particularly in settings characterized by high transmission rates, frequent reinfections, and increased parasite burdens (14). Previous studies by our group have demonstrated that DCs are primarily responsible for an initial T cell activation. Depletion of DCs led to a reduced antigen-specific T cell response in *P. yoelii-*infected animals (15). However, considerable T cell activation could still be observed despite the absence of DCs, suggesting that other APCs, such as macrophages, may compensate for the loss of DCs. Innate immune responses driven by DCs, monocytes, and macrophages are critical during *Plasmodium* infection. The infection triggers the differentiation of monocytes into either DCs or macrophages. Additionally, tissue macrophages, such as those in the spleen, become key players by directly interacting with *Plasmodium* (16). They exhibit a diverse range of responses to malaria, including cytokine secretion and receptor expression, which can be either protective or pathogenic. The complex interplay of these responses is crucial in determining the course of the disease (17).

The spleen is a complex and highly organized organ, which comprises different functional compartments. The red pulp (RP) is the major part of splenic tissue and contains RPMΦ, which efficiently remove dysfunctional, infected and aged RBCs. The marginal zone (MZ) contains marginal zone macrophages (MZMΦ) and marginal metallophilic macrophages (MMMΦ) on the inner border of the MZ, facing the white pulp (WP). Together, these populations delineate the borders of the MZ, facilitating the filtration of blood as it enters this region (18). The MZ also acts as a site for lymphocytes to exit the circulation and migrate into the WP. Within the WP, naïve and central memory T cells are activated by specific antigens, triggering T cell-dependent B cell germinal center reactions and subsequent antibody production (19). MMMΦ and MZMΦ are known for their phagocytic activity of circulating pathogens (20). Especially, MMMΦ play a vital role in immune surveillance, as they are strategically located to effectively capture blood-borne pathogens and antigens and modulate subsequent adaptive immune responses through trophic interactions with neighboring cells (21).

However, studies investigating the role of splenic macrophages during *Plasmodium* infection have yielded conflicting results. In an initial study by Couper et al., depletion of macrophages using CL resulted in increased parasite spread (22), whereas Terkawi and colleagues later observed decreased parasitemia (23). To further clarify the function of macrophages during *Plasmodium* infection, we depleted specific splenic macrophage subsets and analyzed their capability to eliminate iRBCs and to contribute to the induction of T cell responses. Strikingly, we identified RPMΦ to be crucial for parasite control and macrophage subsets of the MZ to contribute to CD4^+^ T cell responses during *P. yoelii* infection.

## Materials and methods

### Mice

All mice were on C57BL/6 background and maintained under specific pathogen-free conditions at the Animal Facility of University Hospital Essen. Female C57BL/6 mice were purchased from Envigo Laboratories (Envigo CRS GmbH, Rossdorf, Germany).

iDTRxCD11ccre (B6.Gt(Rosa26)Sortm1(HBEGF)Awai/Tg(Itgax-cre)1-1Reiz) mice, kindly provided by Ari Waisman, Mainz, Germany and CD169DTR (Siglec1tm1(HBEGF)Mtka) mice have been described previously (15, 24). SpiC^flox/flox^ x vav1cre (B6.Cg-Tg(VAV1-cre)1Graf/Mdf/Spictm1Kmm) mice express the cre recombinase in hematopoietic stem cells and contain loxP-flanks at the s*pi-C* transcription factor locus, which controls RPMΦ development and were kindly provided by Prof. Manfred Kopf, ETH Zürich, Switzerland. OTII (Cg-Tg(TcraTcrb)425Cbn/J) mice express a transgenic T cell receptor recognizing the MHC class II (I-Ab)-restricted ovalbumin peptide_323-339_ (25) and were kindly provided by Dr. Tetyana Yevsa, Medical School, Hannover, Germany. To ensure unbiased group allocation, age- and sex-matched animals were randomly assigned to treatment groups. All animal experiments were carried out in accordance with the guidelines of the German Animal Protection Law and were approved by the state authority for nature, environment, and customer protection, North Rhine-Westphalia, Germany.

### Histology

Spleen samples were excised and washed in PBS before being embedded in Tissue-Tek (Sakura Finetek, Tokyo, Japan). The samples were then rapidly frozen in liquid nitrogen and stored at -80°C. Spleen slices were sectioned using a Cryostat microtome (Leica) at a thickness of 8 µm and mounted on HistoBond^®^ microscope slides (Paul Marienfeld GmbH & Co. KG). The sections were subsequently frozen at -80°C for a minimum of 24 hours before staining. Spleen sections were retrieved from the freezer and allowed to dry at room temperature before use. Samples were then fixed in acetone for 10 minutes, followed by two washes with PBS for 3 minutes each. The areas of interest were outlined with a hydrophobic pen, and the sections were washed again in PBS for 3 minutes. To block nonspecific binding sites, samples were incubated in wash buffer (PBS + 2% FCS) for 45 minutes, then washed twice more in PBS for 3 minutes each. Excess buffer was gently removed, and sections were incubated with FITC-conjugated F4/80, APC-conjugated CD169 and PE-conjugated CD209b (all Miltenyi Biotec, Bergisch Gladbach, Germany) for 30 minutes. After incubation, the sections underwent two additional PBS washes (3 minutes each). Finally, mounting medium and a coverslip were applied.

### *Plasmodium yoelii* infection

For infection, the non-lethal *Plasmodium* strain *P. yoelii* 17XNL was used. Mice were intravenously injected with 1 x 10^5^ *P. yoelii* iRBCs in 100 µl PBS. Parasitemia was assessed on days 3, 5 and 7 p.i. with the Hematology analyzer XN-31 (Sysmex, Kobe, Japan) as described (26).

### Splenic macrophage isolation

To obtain single-cell suspensions from the spleen, the organ was minced into a homogeneous paste before enzymatic digestion (DMEM supplemented with 1 mg/ml Collagenase D and 100 µg/ml DNase) at 37°C for 30 minutes. Following incubation, the cells were mechanically dissociated by passing them through a 100 μm strainer. Erythrocyte lysis buffer was added, and the samples were incubated for 2 minutes at room temperature before being diluted with MΦ medium (DMEM supplemented with 10% FCS and 5 mM EDTA). The suspension was then centrifuged at 300 × g for 10 minutes at 4°C. Finally, the obtained cells were resuspended in PBS supplemented with 2% FCS and 2 mM EDTA for further processing.

### *In vitro* proliferation assay

CD4^+^ T cells were obtained from OT-II mice by rinsing spleens with erythrocyte lysis buffer and washing with PBS supplemented with 2 mM EDTA and 2% FCS. CD4^+^ T cells were isolated from splenocytes by using the CD4^+^ T cell isolation kit (Miltenyi Biotec, Bergisch Gladbach, Germany) according to the manufacturer’s recommendation. DCs were obtained from splenocytes of WT C57BL/6 mice by using CD11c MicroBeads UltraPure (Miltenyi Biotec, Bergisch Gladbach, Germany) according to the manufacturer’s recommendation. Macrophages were isolated as described, and splenic macrophage subpopulations were sorted using an Aria II Cell Sorter (BD Biosciences, Heidelberg, Germany). CD19, NK1.1, Ly-6G, Ly-6C (BD Biosciences, Heidelberg, Germany) CD3 (BioLegend, San Diego, USA) were used as fluorescein isothiocyanate (FITC), CD11b (BioLegend, San Diego, USA) was used as R-Phycoerythrin-Cyanine dye 7 (PE-Cy7), CD169 (BioLegend, San Diego, USA) was used as Allophycocyanine (APC), CD209b (Miltenyi Biotec, Bergisch Gladbach, Germany) was used as R-Phycoerythrin (PE), F4/80 (Invitrogen, Carlsbad, USA) was used as Pacific Blue (PB), and TIM-4 (Invitrogen, Carlsbad, USA) was used as Peridinin chlorophyll protein-Cyanine5.5 (PerCP-Cy5.5). RPMΦ were defined as lineage negative (neg. lin.) (CD3^-^, CD19^-^, LyG^-^, NK1.1^-^), CD11b^int^ and F4/80^+^. MZMΦ were defined as lineage negative (CD3^-^, CD19^-^, LyG^-^, NK1.1^-^), MARCO^+^ TIM4^+^ CD11b^+^, and MMMΦ as lineage negative (CD3^-^, CD19^-^, LyG^-^, NK1.1^-^), CD169^+^ TIM4^+^ CD11b^+^. Once all cell types had been prepared, CD4^+^ T cells were stained with eFluor 670 (eBioscience, ThermoFisher Scientific, Langenselbold, Germany). For this, the cells were washed twice with PBS, resuspended in 2 mL of PBS at room temperature to a concentration of 1 × 10^7^ cells and incubated with eFlour 670 in darkness at 37°C for 10 minutes. Staining was halted by adding 4 mL of cold FCS, followed by an additional 5 minute incubation on ice. Cells were then washed three times with IMDM (supplemented with 10% FCS, 1% Penicillin/Streptomycin and 0.1% Mercaptoethanol), and the supernatant was removed before final resuspension. Cells were plated at a ratio of 2 × 10^4^ APCs to 2 × 10^4^ T cells with 1 µg/ml OVA_323-339_-peptide, and incubated at 37°C. T cell proliferation was assessed after 48 hours incubation. Cells were harvested and analyzed using flow cytometry.

### Antibodies

CD11b, CD62L, CD69 (eBioscience, ThermoFisher Scientific, Langenselbold, Germany) CD19, CD3, CD4, CD86, IL-10, IL-6, Ly-6G, NK1.1, TIM-4 (BD Biosciences, Heidelberg, Germany), CD11c, CD169, CD192, CD3017, CX3CR1, F4/80, IFN-γ, IL-12/IL-23, Ly-6C (BioLegend, San Diego, USA), CD209b (Miltenyi Biotec, Bergisch Gladbach, Germany), granzyme B (Invitrogen, Carlsbad, USA), and MARCO (R&D Systems, Bio-Techne, Wiesbaden, Germany) were used as Allophycocyanine (APC), Brilliant Ultra Violet 737 (BUV737), Brilliant Ultra Violet 805 (BUV805), Horizon V450 (V450), Brilliant Violet 510 (BV510), Brilliant Violet 605 (BV605), Brilliant Violet 711 (BV711), Brilliant Violet 786 (BV786), Fluorescein isothiocynat (FITC), Peridinin-Chlorophyll-Protein (Per-CP), Peridinin chlorophyll protein-Cyanine5.5 (PerCP-Cy5.5), R-Phycoerythrin (PE), R-Phycoerythrin-Cyanine dye 5 (PE-Cy5), R-Phycoerythrin-Cyanine dye 7 (PE-Cy7). Dead cells were identified using the fixable viability dye eFluor 780 (eBioscience, ThermoFisher Scientific, Langenselbold, Germany).

### Flow cytometry

Before surface staining, cells were resuspended in TruStain FcX™PLUS (Biolegend, San Diego, USA) and incubated for 15 minutes on ice. Cells were then centrifuged at 300 x g at 4°C and afterwards stained with the respective surface antibody mix. After surface staining, intracellular staining was performed if applicable. For granzyme B, the Foxp3 staining kit (eBioscience, ThermoFisher Scientific, Langenselbold, Germany) was used following the manufacturer’s instructions. Briefly, cells were fixed in fixation/permeabilization buffer and incubated at RT for 1 hour. Then, cells were permeabilized with permeabilization buffer and centrifuged at 300 x g for 5 min before resuspending cells with the respective intracellular antibodies and incubation for 30 min at 4°C. To assess IFNγ expression, cells were stimulated with 10 ng/mL phorbol 12-myristate 13-acetate and 100 μg/mL ionomycin (both from Sigma-Aldrich, Munich, Germany) for 4 hours in the presence of 5 μg/mL Brefeldin A (Sigma-Aldrich, Munich, Germany). Subsequently, cells were treated with 2% paraformaldehyde and 0.1% IGEPAL CA-630 (Sigma-Aldrich, Munich, Germany) before being stained with the respective antibodies for 30 minutes at 4°C. Flow cytometric analyses were performed using FACS Symphony A5, with data acquisition and analysis conducted using DIVA software (BD Biosciences, Heidelberg, Germany).

### Cell depletion

For the depletion of DCs, iDTRxCD11ccre transgenic (TG) mice were administered diphtheria toxin (DT) (Sigma-Aldrich, Munich, Germany) at a dose of 12 ng/kg body weight in PBS intraperitoneal (i.p.) every other day. As a control group, iDTRxCD11ccre wild-type (WT) littermates received the same DT amount.

CD169^+^ cell depletion was achieved by administration of 30 ng/kg bodyweight DT in PBS i.p. every 5 days. Control groups received PBS i.p. on the same days.

Macrophages were depleted by administration of 200 µl clodronate liposomes (CL) (Liposoma BV, Amsterdam, Netherlands) i.p. every 5 days. Control groups received 200 µl PBS filled liposomes (PBSL) (Liposoma BV, Amsterdam, Netherlands).

### Statistical analysis

Statistical analyses were performed using Graph Pad Prism Software (Graph Pad Software, La Jolla, CA). Gaussian distribution was determined using D’Agostino & Pearson test, Shapiro-Wilk test or Kolmogorov-Smirnov test. If data passed normality test, unpaired Student’s t-test was used, if data were not distributed normally, Mann-Whitney U-test was performed. If two or more groups were to be analyzed, statistical analyses was calculated by one-way ANOVA or Kruskal-Wallis test. Statistical significance was considered as P-values of 0.05 or less (*p < 0.05, **p < 0.01, ***p < 0.001, ****p < 0.0001).

## Results

### DC-depleted mice show decreased CD4^+^ T cell responses during *P. yoelii* infection

Since CD4^+^ T cells play a crucial role in controlling acute infection and establishing long-term immunity, their activation was analyzed upon DC depletion by using DT-treated iDTRxCD11ccre (TG) mice and *P. yoelii* infection. DC depletion was performed one day prior to infection with *P. yoelii*. The mice were sacrificed seven days post-infection (p.i.) to analyze T cell responses and parasitemia as previously described (15). We first confirmed our previous results regarding CD4^+^ T cell activation. Wild-type (WT) mice infected with *P. yoelii* showed a significant rise in CD69^+^ CD4^+^ T cells, indicating enhanced activation (**Supplementary Figure 1**). This increase was also seen in *P.yoelii*-infected DC-depleted transgenic iDTRxCD11ccre mice, although to a significantly lesser extent than in infected WT mice. Accordingly, infected TG mice displayed a marked decrease in CD69-expressing CD4^+^ T cells compared to infected WT mice, suggesting reduced T cell activation in the absence of DCs (**Supplementary Figure 1A**). In agreement with our previous data (15), IFNγ production by CD4^+^ T cells significantly increased upon infection in both WT and TG mice. Interestingly, despite the lack of DCs, DT-treated TG mice showed even higher levels of T cell activation, as evidenced by increased IFNγ-producing CD4^+^ T cells compared to non-infected DT-treated TG mice (**Supplementary Figure 1B**). Upon *P. yoelii* infection the expression of additional T cell activation markers, *i.e.* CD62L and granzyme B, were markedly reduced and increased, respectively (**Supplementary Figures 1C, D**). DT treatment resulted in a slight increase and decrease of CD62L and granzyme B, respectively, in TG mice upon infection, but this effect did not reach significance (**Supplementary Figures 1C, D)**. In summary, we verify that DC-depleted mice show a decrease in CD4^+^ T cell responses during *P. yoelii* infection, but retain substantial activation, which may possibly be due to a compensatory effect of other APCs, such as macrophages.

### Splenic macrophage subpopulations are affected by DC depletion

**Figure 1:**
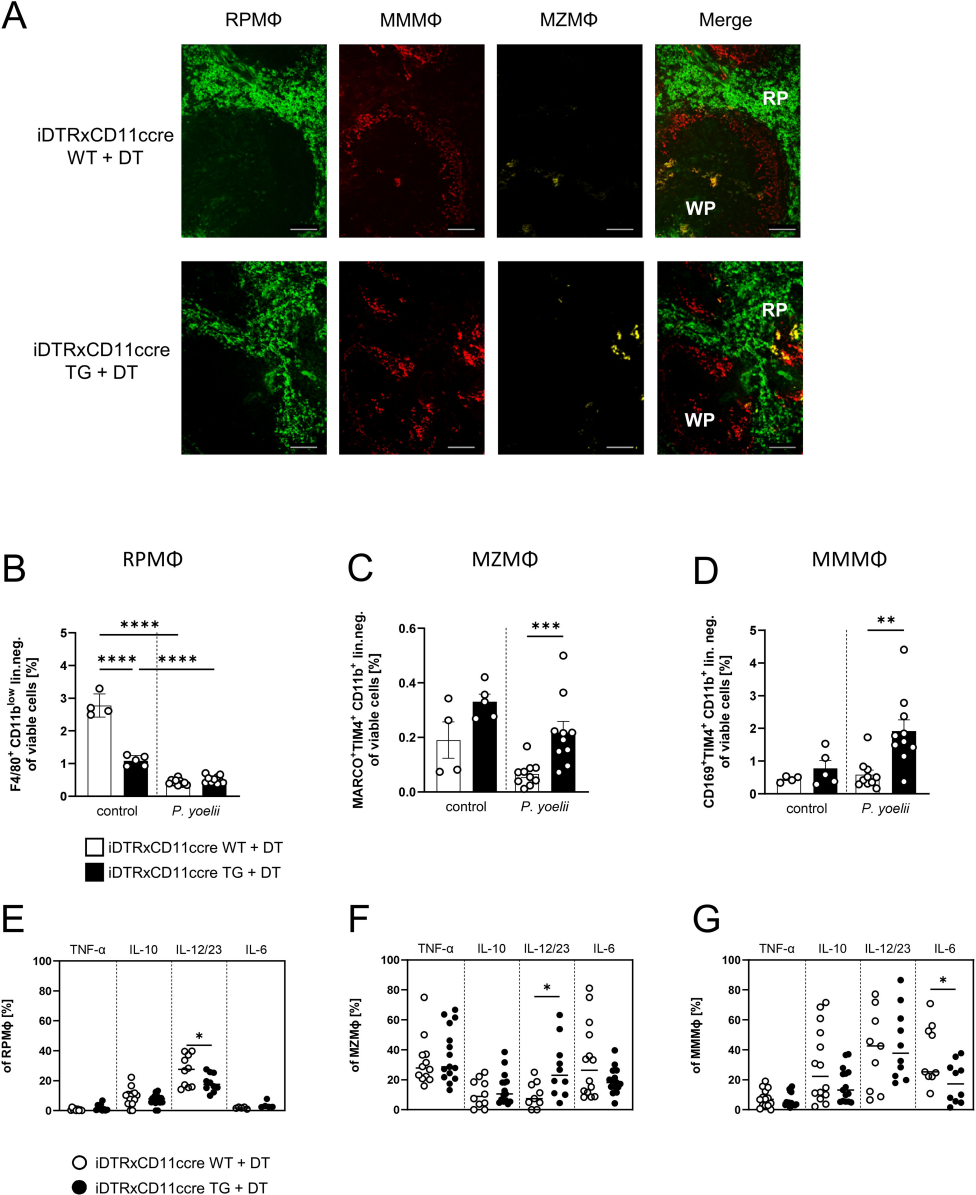
Splenic macrophage subpopulations are affected by DC depletion. **(A)** Splenic tissue of non-infected WT and TG iDTR x CD11ccre mice treated with 12 ng/kg bodyweight DT i.p. for 7 consecutive days was snap-frozen and histologically analyzed. Sections of snap-frozen splenic tissue were analyzed for RPMФ (F4/80^+^; green), MMMФ (CD169^+^; red) and MZMФ (CD209b^+^; yellow). One representative set of n=14 images is shown; scale bar = 100 μm. **(B)** Frequencies of RPMФ (F4/80^+^ CD11b^low^ of neg. lin. **(C)** frequencies of MZMФ (MARCO^+^ TIM4^+^ CD11b^+^ neg.lin.) and **(D)** frequencies of MMMФ (CD169^+^ TIM4^+^ CD11b^+^ neg.lin.) of spleen from uninfected (control) and P. yoelii-infected DT-treated iDTR x CD11ccre WT and TG mice. **(E)** Frequencies of TNFα-, IL-10-, IL-12/23-, and IL-6-expressing RPMФ, **(F)** MZMФ and **(G)** MMMФ in spleen from *P. yoelii*-infected DT-treated iDTR x CD11ccre WT and TG mice were analyzed by flow cytometry 7 days p.i. Results from 3 independent experiments with uninfected (n=4-5) and infected (n=10) are presented as mean (± SEM). Statistical analyses were performed using ordinary one-way ANOVA or Kruskal-Wallis test *p < 0.05, **p < 0.01, ***p < 0.001.

Upon DC depletion, *P. yoelii*-infected animals show an increase in the splenic macrophage compartment (**Supplementary Figure 2D**), indicative of a compensatory mechanisms during hematopoiesis. As the splenic macrophage subsets MZMΦ and MMMΦ rely on the integrity of the MZ structure (27, 28), we first investigated possible effects of DC depletion on the splenic architecture in DT-treated iDTRxCD11ccre mice (TG) and control littermates (WT). Immunofluorescence staining revealed no apparent changes in splenic architecture (**Figure 1A**). The distinction between the WP and RP remained intact in DC-depleted mice. The distribution of RPMΦ (green), MZMΦ (yellow), and MMMΦ (red) was similar between the spleens of DT-treated WT and TG mice, with all macrophages maintaining their typical locations within the spleen. After *P. yoelii* infection, both DT-treated WT and TG mice showed a significant decline in RPMΦ frequency (**Figure 1B**, gating strategy **Supplementary Figure 3**). In contrast, MZMΦ analysis revealed an increase in their abundance in non-infected mice lacking DCs, which became significant following *P. yoelii* infection in DT-treated TG mice (**Figure 1C**). This suggests a significant modulation of MZMΦ population in response to both, DC depletion and infection. Similarly, MMMΦ frequency exhibited a significant rise in DT-treated TG mice compared to WT mice following *P. yoelii* infection (**Figure 1D**). Next, we analyzed the cytokine profile of splenic macrophage subsets from DC-depleted mice after *P. yoelii* infection by flow cytometry and detected significant changes. While the frequency of IL-12/IL-23^+^ RPMΦ from DT-treated TG mice significantly decreased (**Figure 1E**), the percentage of IL-12/IL-23-producing MZMΦ significantly increased upon infection (**Figure 1F**). Additionally, cytokine analysis of MMMΦ showed a significant decrease in IL-6-producing MMMΦ (**Figure 1G**).

These findings suggest an enhanced response of MMMΦ and MZMΦ populations to *P. yoelii* infection in the absence of DCs, possibly indicating altered immune dynamics in this context.

### CD4^+^ T cell activation to *P. yoelii* infection is partly reduced upon macrophage depletion

**Figure 2:**
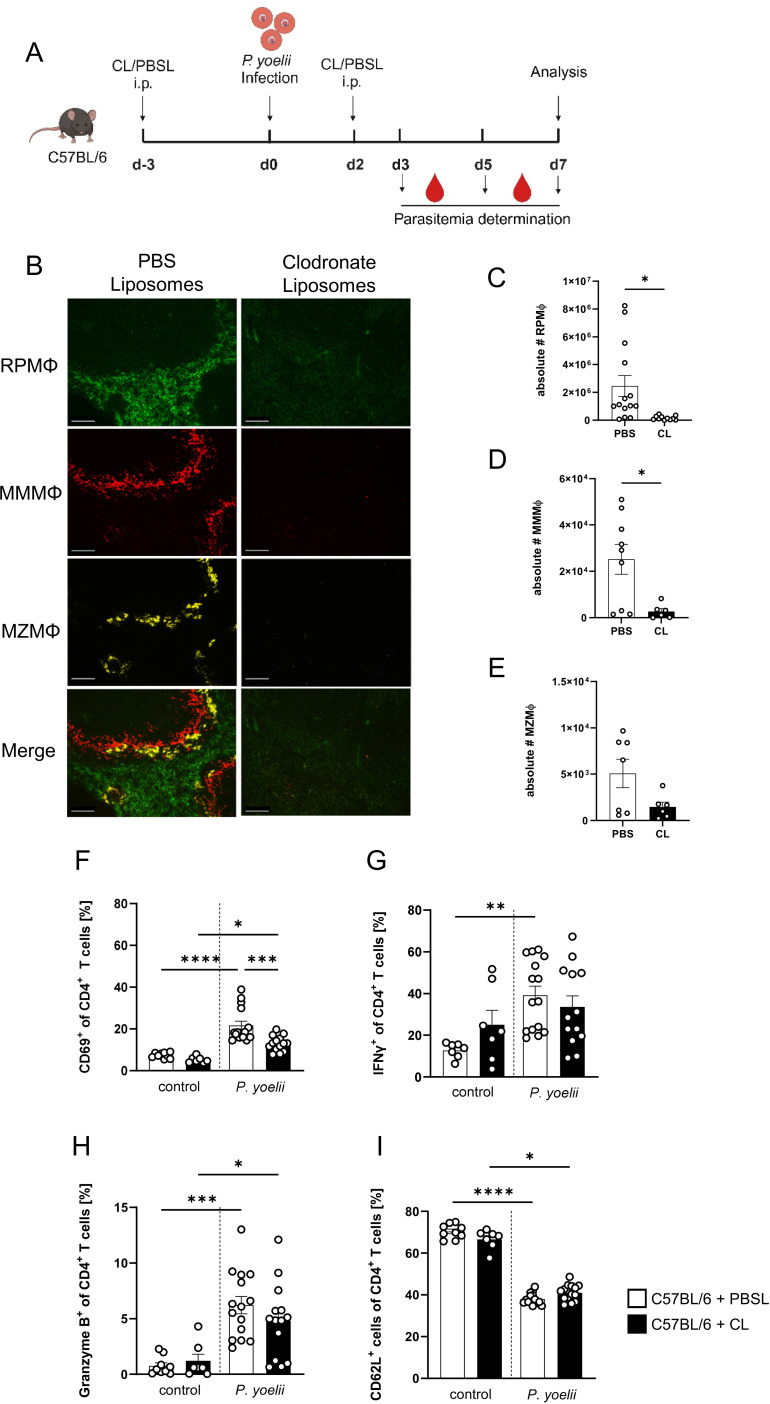
CD4^+^ T cell activation to *P. yoelii* infection is partly reduced upon macrophage depletion. **(A)** Experimental set-up of C57BL/6 mice treated with CL or PBSL. **(B)** Splenic tissue from non-infected C57BL/6 mice treated with either 200 µl PBSL or CL i.p., was snap-frozen and histologically analyzed 10 days post first treatment. Sections of snap-frozen splenic tissue were analyzed for RPMΦ (F4/80^+^; green), MMMΦ (CD169^+^; red) and MZMΦ (CD209b^+^; yellow). One representative set of images is shown (n=7-9); scale bar = 100 µm. Absolute numbers of **(C)** RPMΦ (F4/80^+^ CD11b^low^ of neg. lin.), **(D)** MMMΦ (CD169^+^ TIM4^+^ CD11b^+^ neg. lin.) and **(E)** MZMΦ (MARCO^+^ TIM4^+^ CD11b^+^ neg. lin.), as well as frequencies of **(F)** CD69^+^, **(G)** IFNγ^+^, **(H)** granzyme B^+^ and **(I)** CD62L^+^ cells of CD4^+^ T cells in spleen from uninfected (control) and *P. yoelii*-infected C57BL/6 mice either treated with CL or PBSL were analyzed by flow cytometry 7 days p.i. Results from 3 independent experiments with n=6–15 are presented as mean (± SEM). Statistical analyses were performed using Kruskal-Wallis test or ordinary one-way ANOVA *p < 0.05, **p < 0.01, ***p < 0.001, ****p < 0.0001.

To further investigate the overall impact of splenic macrophages on the outcome of *P. yoelii* infection, we depleted macrophages using the well-established CL model. CL were administered 3 days before *P. yoelii* infection and again 2 days p.i. As a control, PBS-filled liposomes (PBSL) were used, and mice were analyzed 7 days p.i. (**Figure 2A**). The administration of CL resulted in an efficient depletion of all splenic macrophage subsets as confirmed by immunohistochemistry (**Figure 2B**) as well as flow cytometric analysis (**Figures 2C-E**). To ensure that no other phagocytic cells were impacted by CL treatment, various immune cell populations were examined for potential changes in frequency by flow cytometry (**Supplementary Figure 4**). Herein, the neutrophil population was significantly increased, suggesting a possible compensatory effect upon macrophage depletion and cDCs were reduced, but still present. In macrophage-deficient mice infected with *P. yoelii*, a significant reduction in CD69^+^ CD4^+^ T cells was observed (**Figure 2F**). While granzyme B is classically associated with cytotoxic CD8^+^ T cells, it is also expressed by specific CD4^+^ T cell subsets, called cytotoxic Th1 cells. Granzyme B^+^ CD4^+^ T cells appear to participate in anti-*Plasmodium* immunity, but the mechanism remains elusive, making them interesting for our analysis (29). Herein, we observed a slight trend to lower frequencies of IFNγ- and granzyme B-expressing CD4^+^ T cells from the CL-treated, infected group, which did not reach significance (**Figures 2G, H**). In contrast, CD62L levels were not altered in the infected groups, but showed a significant decrease upon *P. yoelii* infection indicating an overall increase in CD4^+^ T cell activation (**Figure 2I**). Together, these results suggest that macrophages contribute, at least in part, to CD4^+^ T cell activation during infection.

### Macrophage depletion results in an impaired parasite clearance

**Figure 3:**
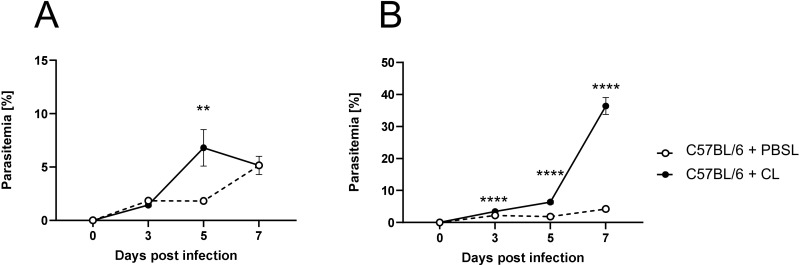
Macrophage depletion results in an impaired parasite clearance. **(A)** C57BL/6 mice were treated with 200 µl of CL or PBSL i.p. 3 days prior to, or **(B)** 2 days post *P. yoelii* infection. Parasitemia was determined with 10 µl blood in a 1/10 dilution using the hematology analyzer Sysmex XN31 3, 5 and 7 days post *P. yoelii* infection in CL- and PBSL-treated C57BL/6 mice. Results from 3 independent experiments with n=8–16 are presented as mean (± SEM). Statistical analyses were performed using unpaired Student’s t-test or Mann-Whitney test **p < 0.01 ****p < 0.0001.

We next quantified parasite blood infection dynamics following macrophage depletion (**Figure 3**). In the first infection experiment, CL administration was initiated three days before *P. yoelii* infection (**Figure 3A**). Here, a significant increase in parasitemia was observed 5 days p.i. in CL-treated mice compared to PBSL-treated controls. However, by day 7 p.i., parasitemia levels in CL-treated mice decreased and resulted in equalized levels between the groups, suggesting that other phagocytic cells may compensate for the loss of macrophage function. To determine whether this equalizing effect occurs when macrophages are depleted after an infection is established, C57BL/6 mice were first infected with *P. yoelii* and treated with PBSL or CL starting 2 days p.i. (**Figure 3B**). Interestingly, in this experimental setting a significant increase in parasitemia was detected 24 hours later (3 days p.i.) in CL-treated mice. This exacerbation became even more pronounced by day 5 p.i. and peaked at an excessively high parasitemia of approximately 40% by day 7 p.i. (**Figure 3B**). Together, the altered blood infection dynamics in CL-treated mice corroborate the notion that macrophages are the main cells essential for parasite clearance during *P. yoelii* infection.

### MMMΦ are involved in CD4^+^ T cell effector function, but are dispensable for parasite clearance

Given that the CL treatment depletes all splenic macrophage subsets, the next step was to identify which macrophage subset might be responsible for the reduced CD4^+^ T cell activation and uncontrolled parasite burden observed during *P. yoelii* infection in macrophage-depleted mice. To determine whether different splenic macrophage populations can generally induce a T cell response, we first analyzed their T cell activating capacity *in vitro.* Therefore, OVA_323-339_-peptide was loaded on sorted splenic macrophage subpopulations as well as on DCs, which were co-cultured with eFluor670-labeled OVA-specific CD4^+^ T cells. T cell proliferation was then assessed by the dilution of the proliferation dye (**Supplementary Figure 5**). All splenic macrophage subpopulations were capable of inducing an OVA-specific T cell proliferation, with RPMΦ and MMMΦ demonstrating the strongest potential, followed by MZMΦ. These results suggest that splenic macrophages can elicit T cell responses at least *in vitro*.

**Figure 4:**
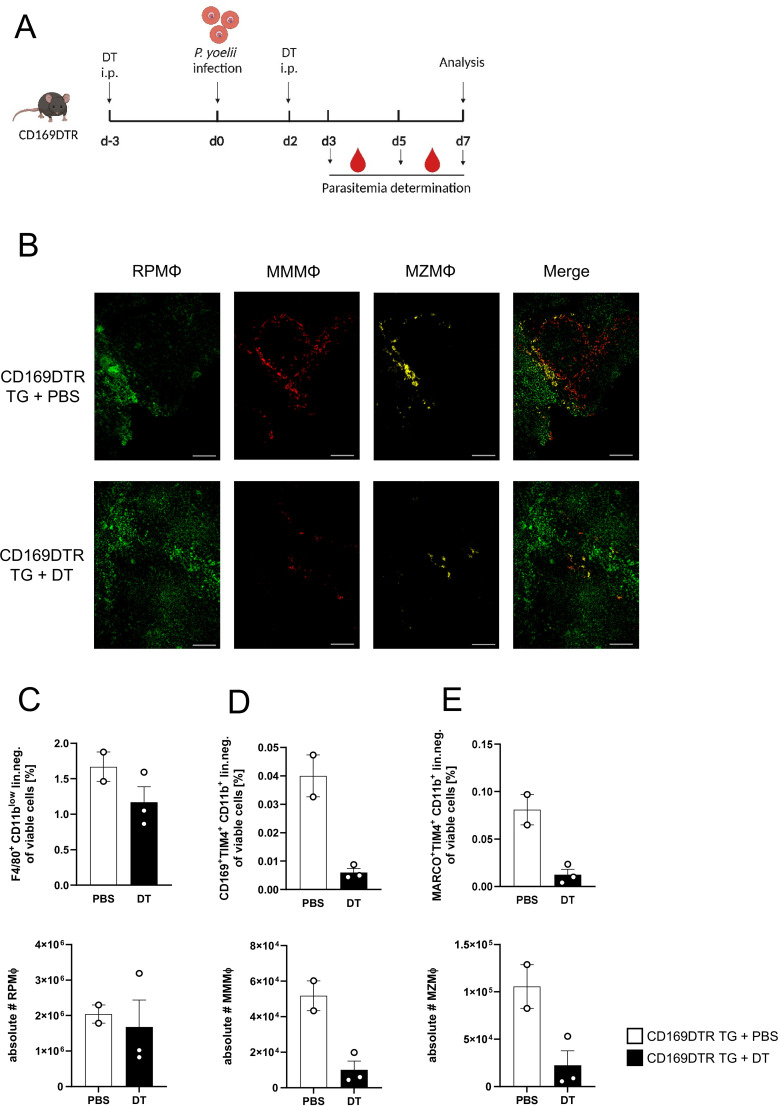
Efficient depletion of MMMΦ and MZMΦ in DT-treated CD169DTR mice. **(A)** Experimental set-up of CD169DTR mice treated with DT or PBS. **(B)** Splenic tissue of non-infected CD169DTR mice treated with DT or PBS i.p. analyzed 10 days post first treatment was snap-frozen and histologically analyzed. Sections of snap-frozen splenic tissue were analyzed for RPMΦ (F4/80^+^; green), MMMΦ (CD169^+^; red) and MZMΦ (CD209b^+^; yellow). One representative set of images is shown (n=7-9); scale bar = 100 µm. Frequencies (top) and absolute cell numbers (bottom) of **(C)** RPMΦ (F4/80^+^ CD11b^int^ neg. lin.), **(D)** MMMΦ (CD169^+^ TIM4^+^ neg. lin.) and **(E)** MZMΦ (MARCO^+^ TIM4^+^ neg. lin.) of spleen from uninfected CD169 mice either treated with DT or PBS for 10 days were analyzed via flow cytometry. Results from 1 experiment with n=2–3 are presented as mean (± SEM).

**Figure 5:**
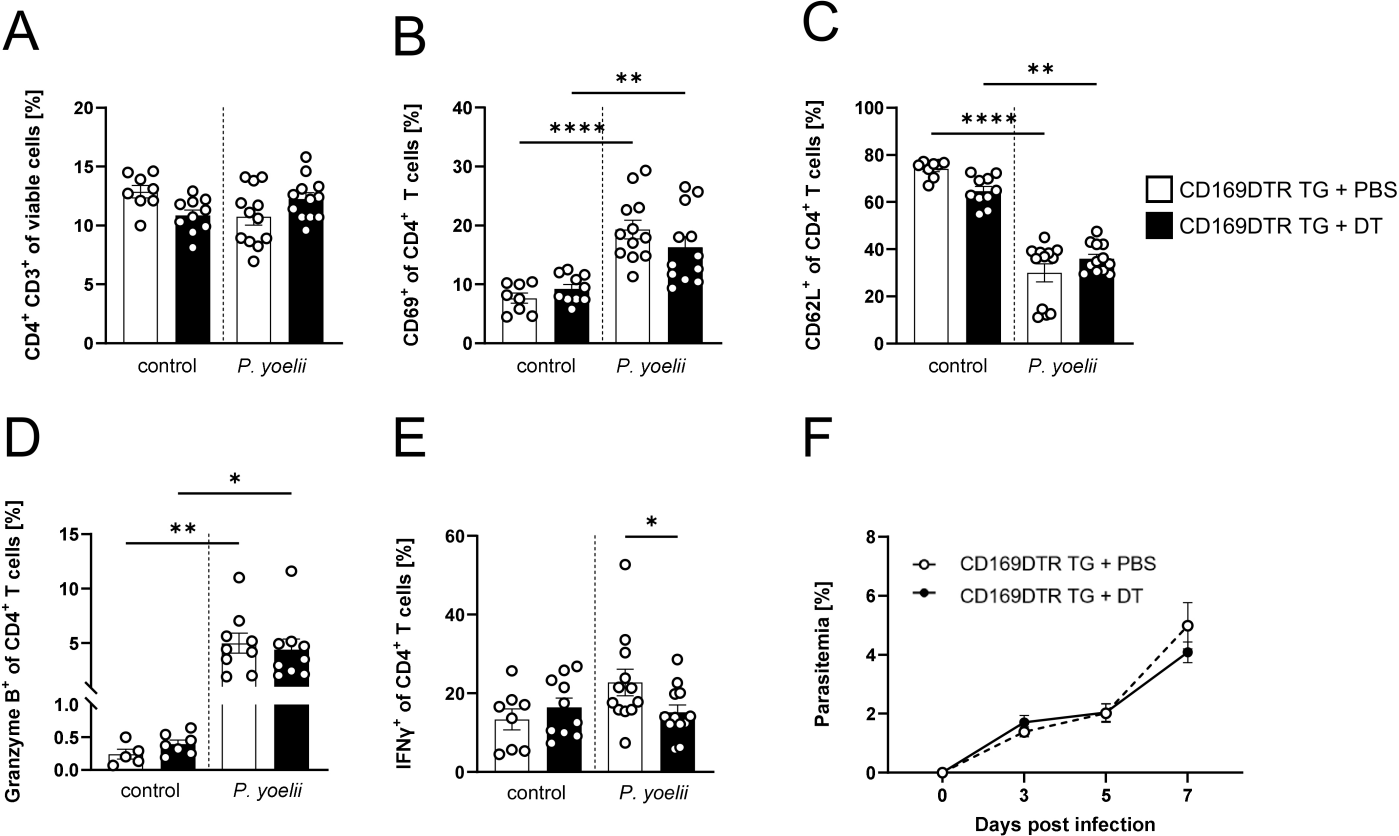
MMMΦ are involved in CD4^+^ T cell effector function, but are dispensable for parasite clearance. **(A)** Frequencies of CD4^+^ T cells **(B)** CD69^+^ of CD4^+^ T cells, **(C)** CD62L^+^ of CD4^+^ T cells, **(D)** granzyme B^+^ of CD4^+^ T cells and **(E)** IFNγ^+^ of CD4^+^ T cells in spleen from uninfected (control) and *P. yoelii*-infected CD169DTR mice either treated with DT or PBS, were analyzed by flow cytometry 7 days p.i. Results from 3 independent experiments with n=8–12 are presented as mean (± SEM). Statistical analyses were performed using ordinary one-way ANOVA or Mann-Whitney Test *p < 0.05, **p < 0.01, ****p < 0.0001. **(F)** Parasitemia was determined with 10 µl blood in a 1/10 dilution using the hematology analyzer Sysmex XN31 3, 5 and 7 days post *P. yoelii* infection in CD169DTR mice treated either with 30 ng/kg bodyweight DT or PBS as control. Results from 4 independent experiments with n=18–20 are presented as mean (± SEM). Statistical analyses were performed using unpaired Student’s t-test or Mann-Whitney test.

Since a significant increase in MMMΦ was observed during *P. yoelii* infection in DC-depleted mice (**Figure 1D**), along with their potential to induce CD4^+^ T cell proliferation *in vitro* (**Supplementary Figure 5**), this macrophage subset was of particular interest for further analysis. Therefore, CD169DTR mice were used to specifically deplete MMMΦ *via* diphtheria toxin (DT). DT treatment started at day 3 prior to *P. yoelii* infection and was repeated every 5 days. Parasitemia was monitored on days 3, 5, and 7 p.i., and mice were sacrificed for analysis 7 days p.i. (**Figure 4A**). While DT administration in CD169DTR mice did not alter splenic architecture (**Figure 4B**), or other immune cells (**Supplementary Figure 6**), it resulted in a reduction in MMMΦ, and to a lesser extent, MZMΦ, as determined by immunofluorescence analysis (**Figure 4B**) and confirmed by flow cytometry (**Figures 4C-E**). To assess the effect of MMMΦ and MZMΦ depletion on CD4^+^ T cells, we analyzed the T cell response in *P. yoelii-*infected DT-treated CD169DTR mice. We did not detect differences in CD4^+^ T cell frequencies following DT treatment and *P. yoelii* infection (**Figure 5A**). Moreover, the expression of activation markers, *i.e.* CD69, CD62L, and granzyme B, did not differ between MMMΦ-depleted and control mice after infection (**Figures 5B-D**). Of note, MMMΦ and MZMΦ depletion led to a slight, but significant, reduction in IFNγ^+^ CD4^+^ T cells at 7 days p.i. (**Figure 5E**). However, depletion of MMMΦ and MZMΦ did not affect the course of parasitemia until day 7 p.i. (**Figure 5F**), excluding an important role in phagocytosis of parasite-infected erythrocytes.

In conclusion, MMMΦ and MZMΦ contribute to the induction of a T_H_1 response, but their contribution to clear *Plasmodium*-infected RBCs is likely negligible.

### RPMΦ are indispensable for parasite clearance, but not essential for a functional CD4^+^ T cell response

**Figure 6:**
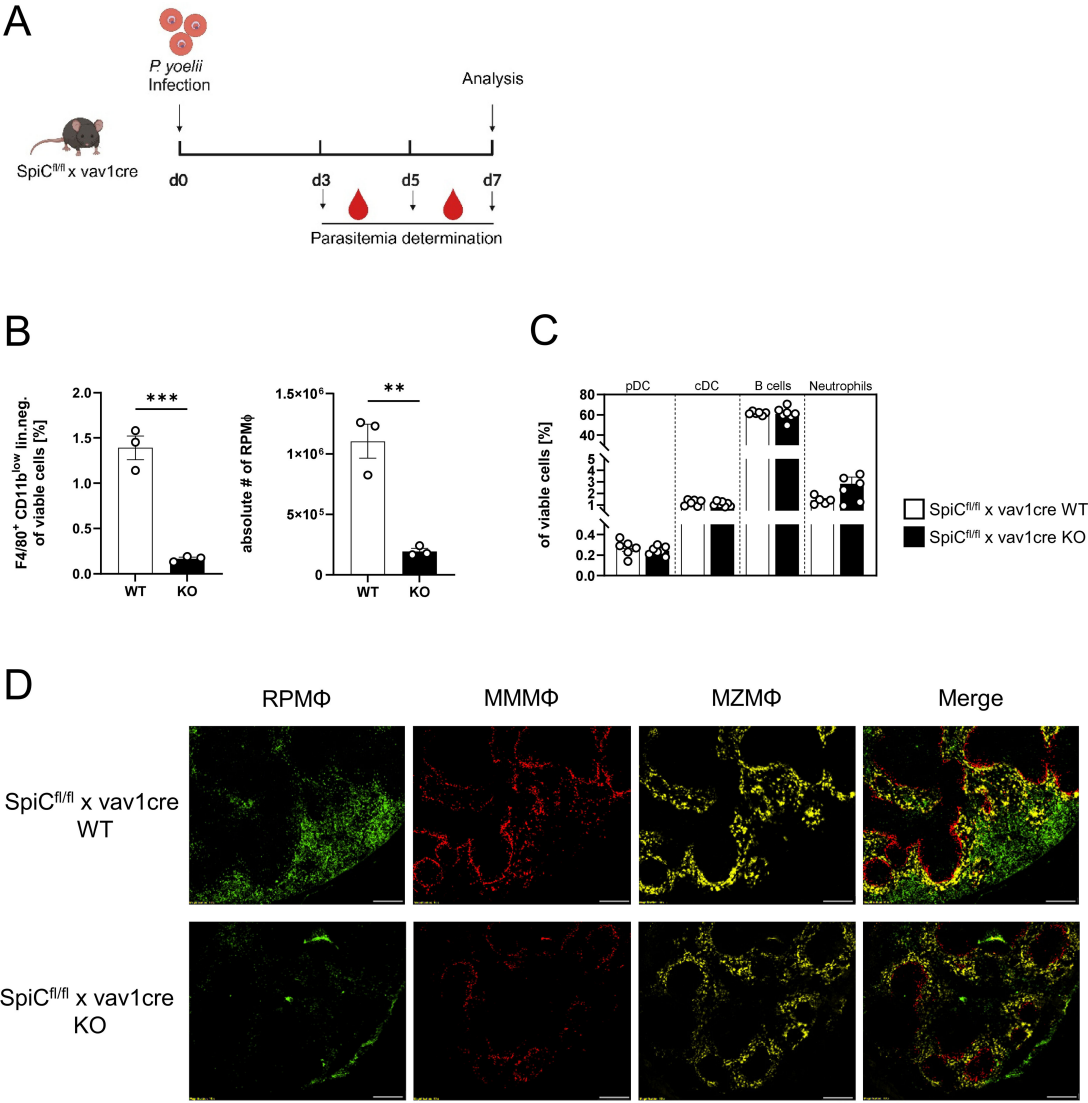
Effective depletion of RPMΦ in Spi-C KO mice. **(A)** Experimental set-up of SpiC^flox/flox^ x vav1cre mice. **(B)** Frequencies of RPMΦ (F4/80^hi^ CD11b^low^ neg. lin.) and **(C)** pDCs, cDCs, B cells and neutrophils in spleen of SpiC^flox/flox^ x vav1cre (cre negative = WT, cre positive = KO) were analyzed by flow cytometry 7 days p.i. Results from 1–2 experiments with n=3–6 are presented as mean (± SEM). Statistical analyses were performed using Mann-Whitney test **p < 0.01, ***p < 0.001. **(D)** Splenic tissue of non-infected SpiC^flox/flox^ x vav1cre mice was snap-frozen and histologically analyzed. Sections of snap-frozen splenic tissue were analyzed for RPMΦ (F4/80^+^; green), MMMΦ (CD169^+^; red) and MZMΦ (CD209b^+^; yellow). One representative set of images is shown (n=6); scale bar = 100 µm.

**Figure 7:**
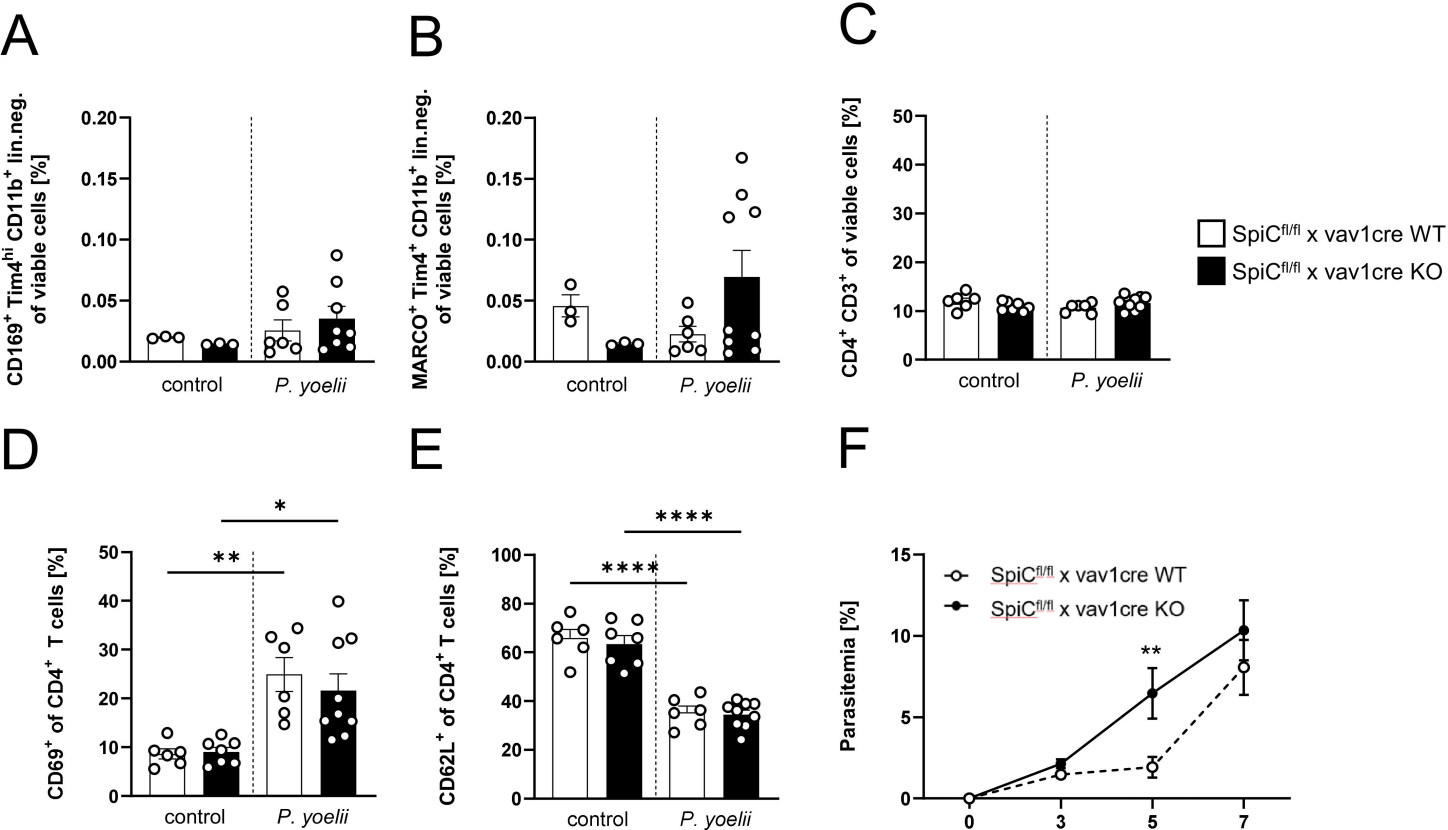
RPMΦ are indispensable for parasite clearance, but not essential for a functional CD4^+^ T cell response. **(A)** Frequencies of MMMΦ (CD169^+^ TIM4^+^ of CD11^+^ neg. lin.), **(B)** MZMΦ (MARCO^+^TIM4^+^ of CD11b^+^ neg. lin.) and **(C)** CD4^+^ T cells, as well as frequencies of **(D)** CD69^+^ and **(E)** CD62L^+^ of CD4^+^ T cells in spleen from uninfected (control) and *P. yoelii*-infected SpiC^flox/flox^ x vav1cre mice (cre negative = WT, cre positive = KO) were analyzed by flow cytometry 7 days p.i. Results from 2 experiments with n=3–9 are presented as mean (± SEM). Statistical analyses were performed using Mann-Whitney test *p < 0.05, **p < 0.01, ****p < 0.0001. **(F)** Parasitemia was determined with 10 µl blood in a 1/10 dilution using the hematology analyzer Sysmex XN31 3, 5 and 7 days post *P. yoelii* infection in KO and WT SpiC^flox/flox^ x vav1cre mice (cre negative = WT, cre positive = KO). Results from 3 independent experiments with n=5–12 are presented as mean (± SEM). Statistical analyses were performed using unpaired Student’s t-test or Mann-Whitney test **p < 0.01.

Since the general depletion of splenic macrophages in *P. yoelii*-infected mice by CL treatment resulted in a significant increase in parasitemia (**Figures 3A, B**), but MMMΦ and MZMΦ depletion did not significantly alter parasite burden, we next analyzed the impact of RPMΦ on clearing *P. yoelii*-infected RBCs. We used SpiC^flox/flox^ x vav1cre mice, a transgenic mouse model in which RPMΦ development is selectively controlled. KO mice and WT littermates were infected with *P. yoelii* and analyzed 7 days p.i. (**Figure 6A**). Indeed, we could demonstrate, a significant reduction of the RPMΦ population in SpiC^flox/flox^ x vav1cre KO mice compared to SpiC^flox/flox^ x vav1cre WT littermates (**Figures 6B, D**), while pDCs, cDCs, B cells, and neutrophil frequencies are not altered due to the RPMΦ deficiency (**Figure 6C**). We note that MZMΦ and MMMΦ showed a trend towards increased frequencies, which did not reach significance in infected KO mice compared to WT mice (**Figures 7A, B**). The absence of RPMΦ did not impact the CD4^+^ T cell response during *P. yoelii* infection, as no notable differences in CD4^+^ T cell frequencies and activation status were found between WT and KO mice upon infection (**Figures 7C-E**). This implicates, that the RPMΦ subpopulation does not contribute to CD4^+^ T cell priming. However, RPMΦ deficiency resulted in significant elevated parasitemia at 5 days p.i. (**Figure 7F**). This impairment of parasite clearance is comparable to the findings we obtained after depleting all macrophages by CL. In summary, the data suggest that RPMΦ are not essential for the induction of CD4^+^ T cell responses during *P. yoelii* infection, but were identified as being important for clearing *P. yoelii* iRBCs.

## Discussion

Macrophages are well known to act as phagocytic cells that form the first line of defense by eliminating invading pathogens. During malaria, splenic macrophages protect the host through cytokine production and phagocytosis (17). Previously, we demonstrated that depletion of DCs resulted in impaired T cell responses, but residual T cell activation was still present, and the infection was ultimately cleared, with its course remaining unchanged (15). These findings indicate that although DCs are vital for optimal T cell activation, their function can be partially replaced by other cells during *P. yoelii* infection (15, 30). Therefore, we analyzed the potential of different splenic macrophage subsets to initiate T cell responses in addition to their capacity to eliminate iRBCs during an ongoing *P. yoelii* infection. Our data show that *P. yoelii* infection of DC-deficient mice resulted in elevated frequencies of MZMΦ and MMMΦ, whereas the relative number of RPMΦ decreased in both, DC-deficient as well as WT mice, during infection. A reduction in RPMΦ following *Plasmodium* infection has been demonstrated previously and was attributed to the phagocytosis of iRBCs. This in turn results in ferriprotoporphyrin (heme) accumulation, a by-product of hemoglobin digestion, which triggers apoptosis in a dose-dependent manner (31). While, we cannot exclude the possibility that other mechanisms contribute to the decrease in RPMΦ, it is plausible that the RPMΦ reduction observed in our analyses is also caused by ferriprotoporphyrin accumulation in the cells. The frequency of RPMΦ from *P. yoelii*-infected mice lacking DCs that express the proinflammatory cytokines IL-12/23 was significantly reduced, whereas relative numbers of IL-12/23-producing MZMΦ was upregulated. These cytokines are crucial for initiating T_H_1 and T_H_17 responses (32). Especially T_H_1 cells are of high importance for the control of intracellular parasites, due to their role in inducing a protective immune response by IFNγ production (12). Although the cytokine profile of MMMΦ was not significantly altered, we detected an almost 4-fold increase in relative numbers of MMMΦ in DC-depleted *P.yoelii*-infected mice. These results indicate that DC depletion leads to changes in splenic macrophage subpopulations upon *P. yoelii* infection, and that these may therefore be involved in T cell activation, at least in the absence of DCs. To further clarify the role of splenic macrophages on the CD4^+^ T cell response during *P. yoelii* infection, we depleted macrophages by using CL. As expected, treatment with CL resulted in efficient depletion of all splenic macrophage subsets. In accordance with a previous study by Rooijen et al., neutrophils and DCs in the spleen are not depleted by intraperitoneal (i.p.) CL treatment (33), although cDCs are slightly reduced, but still present. We also observed a significant increase in neutrophils upon macrophage depletion. In fact, neutrophils have been described to be involved in the defensive response against *Plasmodium* by producing reactive oxygen species and directly phagocytosing the parasite and might therefore compensate for the lack of macrophages. However, they might also contribute to pathogenesis of severe malaria due to release of toxic granule proteins and neutrophil extracellular traps (34).

Analysis of CD4^+^ T cell activation markers revealed significantly decreased frequencies of CD69^+^ CD4^+^ T cells in CL-treated compared to PBSL-treated mice during *P. yoelii* infection. The observed reduction in CD69, an early activation marker, suggests that macrophages may contribute to the initial activation phase of CD4^+^ T cells to some extent, potentially via cytokine release or co-stimulatory interactions, even in the presence of cDCs in our model.

To elucidate which specific splenic macrophage subsets might be responsible for this, we decided to use CD169DTR mice. DT treatment of these mice resulted not only in depletion of CD169^+^ MMMΦ, but also affected MZMΦ in our hands. Importantly, DC subsets, neutrophils and B cells were not affected by DT treatment, confirming the specificity of this mouse model. In the spleen, MMMΦ are strategically located at the MZ, near areas rich in T and B lymphocytes, allowing them to efficiently interact with body fluids and perform their roles in antigen uptake and processing. This positioning supports their involvement in coordinating immune responses (35, 36). Our work provides evidence, that all tested macrophage subsets, but especially MMMΦ, are capable to induce CD4^+^ T cell proliferation *in vitro*. Strikingly, we detected a significantly reduced relative number of CD4^+^ T cells producing IFNγ in addition to a partial reduction of CD69^+^ and granzyme B^+^ CD4^+^ T cells in DT-treated and *P. yoelii*-infected CD169DTR mice, suggesting that MMMΦ and/or MZMΦ contribute, at least in part, to the activation of CD4^+^ T cells during infection. It is well known that IFNγ, produced by T_H_1-polarized CD4^+^ T cells, plays a central role in controlling *Plasmodium* blood-stage infection. It enhances macrophage effector functions and contributes to parasite clearance. In this light, the significant reduction in IFNγ expression observed in CD4^^+^^ T cells from CD169DTR mice underscores the functional contribution of CD169^^+^^ macrophages to the T_H_1 response. Although no additional activation markers reached significance, this cytokine-specific reduction still suggests a dampened immune environment in the absence of MZMΦ and MMMΦ. While DCs take over the primary role in CD4^^+^^ T cell activation in our model, the observed effects point to an auxiliary role for macrophages. This aligns with recent work suggesting that CD169^^+^^ macrophages may serve as alternative platforms for antigen presentation, especially when conventional DC function is impaired (35). This finding has important implications for human malaria, where DC dysfunction has been observed during blood-stage infections, particularly in individuals from high transmission areas with frequent reinfections and elevated parasite burdens (14).

Previous work showed that absence of MMMΦ in *Plasmodium berghei ANKA* (*PbA*)-infected mice causes a marked increase in parasite sequestration, resulting in vascular occlusion, leakage, and enhanced tissue deposition of hemozoin, which ultimately leads to extensive tissue damage and widespread inflammation affecting multiple organs (37). Therefore, an intact layer of MMMΦ is crucial for pathogen clearance and disease prevention, as they help trigger a rapid and strong innate immune response (38). However, when parasitemia was measured in CD169DTR mice, no differences were found between DT-treated and PBS-treated mice, indicating that this macrophage subset is not directly responsible for clearing iRBCs during *P. yoelii* infection. Nevertheless, depletion of all macrophages by using CL resulted in a significant increase in parasitemia at day 5 after *P. yoelii* infection, but returned to control levels at day 7. Since CL depletes both macrophages and circulating monocytes, cells that could potentially repopulate the spleen and contribute to the immune response against *Plasmodium* (33), the normalization of parasitemia might be explained by an increase in neutrophils, which are capable to phagocytose iRBCs and of generating reactive oxygen species (ROS) to combat the infection (39).

As macrophage depletion began prior to infection, compensatory mechanisms may have been activated early, potentially leading to the observed equilibrium of parasitemia between CL-treated and control mice. To investigate whether macrophages are primarily responsible for removal of iRBCs, additional experiments depleting macrophages 2 days after infection were performed, ensuring that other cell populations could not compensate for the loss of macrophages beforehand. In this case, a significant increase in parasitemia was detected 3 days post infection in CL-treated mice. This effect became more pronounced on day 5, with parasitemia peaking at an unusually high 40% by day 7, further confirming that macrophages are essential for the clearance of iRBCs.

Since our results from DT-treated *P. yoelii*-infected CD169DTR mice provide evidence that MMMΦ and MZMΦ are not essential for effective pathogen clearance, we hypothesized that RPMΦ were responsible for impaired parasite clearance in CL macrophage-depleted mice. RPMΦ primarily function by phagocytosing aged and damaged erythrocytes as they pass through the RP (17, 18, 40). In mice, RPMΦ uniquely express the transcription factor Spi-C, which is necessary for their development (41, 42). Therefore, it is possible to deplete them specifically by eliminating Spi-C expression in all nucleated blood cells and hematopoietic stem cells in SpiC^flox/flox^ x vav1cre transgenic mice (Spi-C KO). CD4^+^ T cell activation showed no significant changes in Spi-C KO mice upon infection. Interestingly, analysis of parasitemia in Spi-C KO mice, revealed a significant increase 5 days p.i., followed by a normalization by day 7, closely mirroring the results seen in mice that were treated with CL before infection. These findings confirm that RPMΦ are critical for the clearance of parasites, but are not involved in CD4^+^ T cell priming during *P. yoelii* infection. The eventual normalization of parasitemia could be again explained by the activity of other phagocytic cells, such as neutrophils, which may help control the infection in the absence of RPMΦ. Further experiments are warranted to study the redundancy of phagocytic cells in malaria control. In addition, a temporal comparison to better delineate the phagocytic capacity of RPMΦ during both, early and late stages of infection would be interesting.

In our current study, we analyzed macrophage subsets in their native microanatomical niches within the spleen. These subsets, namely RPMΦs, MMMΦs, and MZMΦs are each localized to distinct and well-defined anatomical regions (red pulp, marginal zone, and marginal metallophilic zone, respectively). Importantly, all analyzed macrophage subsets are tissue-resident macrophages that populate the spleen during embryonic development and are maintained locally from birth onward. Their distinct localization is a key reason why they are particularly interesting to study in the context of malaria, as their positioning likely correlates with distinct functional roles during infection. Generally, macrophage plasticity, particularly under conditions of depletion or inflammation, represents an important future direction. In particular since monocytes are known to repopulate the spleen and differentiate into macrophages under inflammatory or depletion conditions, which could restore at least some of the lost functions (43). Indeed, it has been shown that spleen and liver macrophages are repopulated by inflammatory monocytes after macrophage depletion during blood-stage malaria (43). In our targeted depletion experiments the analyzed macrophage subsets with their defined surface molecule expression were still depleted in the respective models. Therefore, it is unlikely that repopulating monocytes or macrophages re-differentiate into the same subset identities (e.g., true MMMΦs or MZMΦs) with identical molecular phenotypes. However, we cannot rule out the possibility that the function of these subpopulations is taken over by other cells with different surface markers, which we did not include in our analysis. Accordingly, it would be interesting to examine, whether repopulating monocytes or macrophages take over or merely adopt similar functional profiles of the specific macrophage subpopulations in a future project.

In summary, our data support the concept that macrophages can compensate for DC deficits. The identification that the functionally distinct macrophage subset of RPMΦ is primarily involved in parasite clearance contributes to our understanding in the compartmentalization and flexibility of macrophage function during blood-stage infection. Specific targeting of these subsets could open up new therapeutic strategies to control parasite propagation and aid vaccine efficacy.

## Data Availability

The raw data supporting the conclusions of this article will be made available by the authors, without undue reservation.
