# Supplementary material for: Red pulp macrophages clear parasites, while marginal metallophilic and marginal zone macrophages support CD4+ T cell activation during Plasmodium yoelii infection
Source: Front Immunol. 2025 Jul 17;16:1607201. doi: 10.3389/fimmu.2025.1607201 (PMC12310687; doi:10.3389/fimmu.2025.1607201)
Supplement: Supplementary file 1 [file DataSheet1.docx]

Supplementary Material


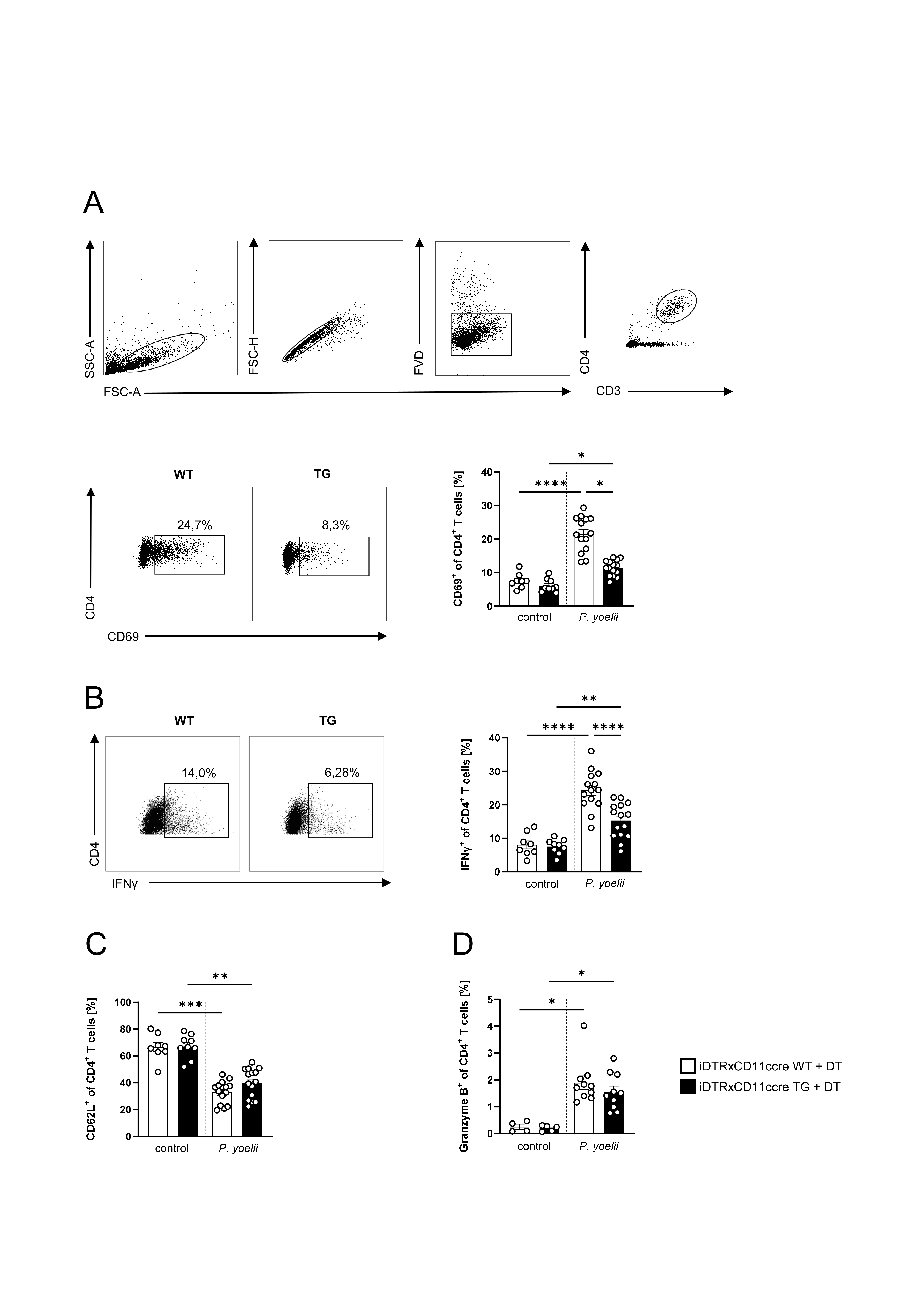


Supplemental Figure 1: DC-depleted mice show decreased CD4^+^ T cell responses during *P. yoelii* infection

(**A**) Representative dot plots of isolated splenocytes and CD4^+^ CD3^+^ T cells that were analyzed via flow cytometry, as well as representative dot plot of CD69^+^ CD4^+^ T cells and summarized frequencies of CD69^+^ CD4^+^ T cells. (**B**) Representative dot plot of IFNγ^+^ CD4^+^ T cells and frequencies of IFNγ^+^ CD4^+^ T cells, (**C**) frequencies of CD62L^+^ CD4^+^ T cells and (**D**) granzyme B^+^ CD4^+^ T cells in spleen from uninfected (control) and *P. yoelii*-infected DT-treated iDTR x CD11ccre WT and TG mice that were analyzed by flow cytometry 7 days p.i. Results from 3 independent experiments with n=6-15 are presented as mean (± SEM). Statistical analyses were performed using Kruskal-Wallis test or ordinary one-way ANOVA **p* < 0.05, ***p* < 0.01, ****p* < 0.001, *****p* < 0.0001.


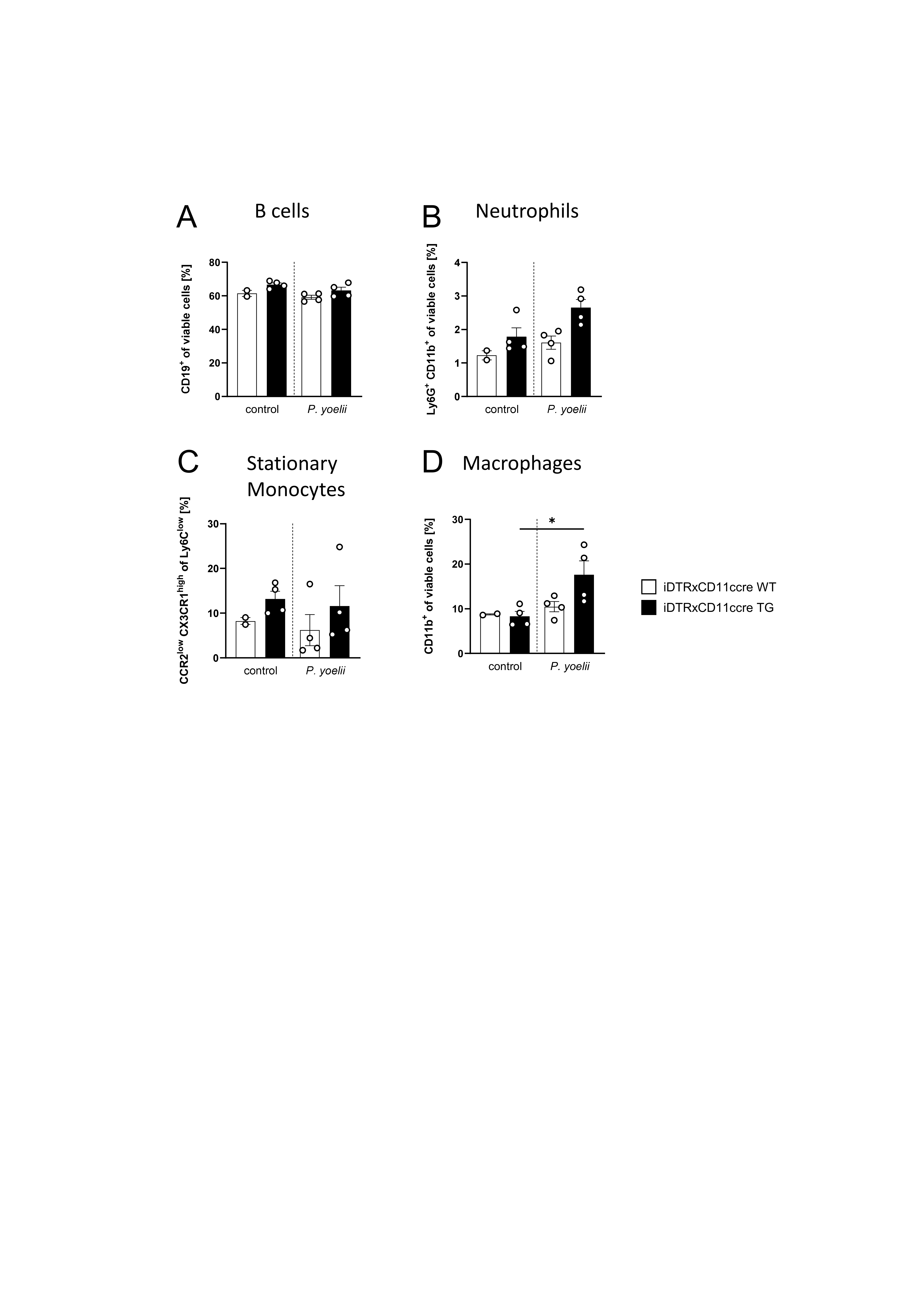


Supplemental Figure 2: DC depletion leads to an expansion of the splenic macrophage compartment

(**A**) Frequencies of CD19^+^ B cells, (**B**) Ly6G^+^ CD11b^+^ neutrophils, (**C**) CCR2^low^ CX3CR1^high^ of Ly6C^low^ cells presenting stationary monocytes and (**D**) CD11b^+^ cells in spleen from uninfected (control) and *P. yoelii*-infected DT-treated iDTR x CD11ccre WT and TG mice 3 days p.i. Results from 1 experiment with n=2-4 are presented as mean (± SEM). Statistical analysis was performed using unpaired Kruskal-Wallis test **p* < 0.05.


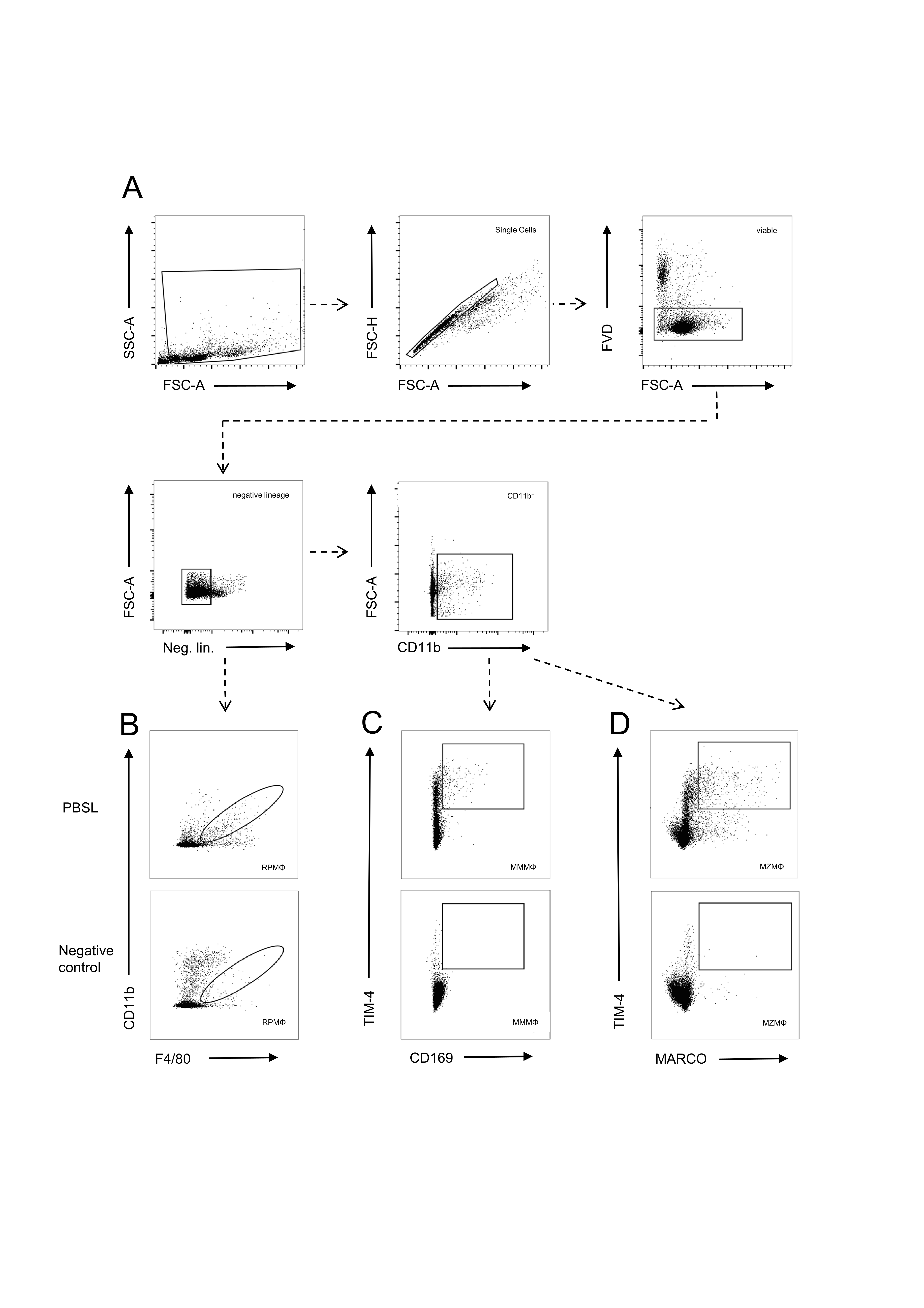


Supplemental Figure 3: Gating strategy of splenic macrophage subpopulations

(**A**) Representative flow cytometry gating strategy of splenic macrophage subpopulations of C57BL/6 WT mice treated with PBSL or CL as negative control from all cells to single and viable cells defined as FVD^-^. (**B**) RPMΦ were defined as lineage negative (CD3^-^ CD19^-^ NK1.1^-^ Ly6G^-^) and F4/80^+^ CD11b^low^ (**C**) MMMΦ were defined as lineage negative, CD11b^+^ TIM-4^+^ CD169^+^ and (**D**) MZMΦ were defined as lineage negative, CD11b^+^ TIM-4^+^ MARCO^+^.


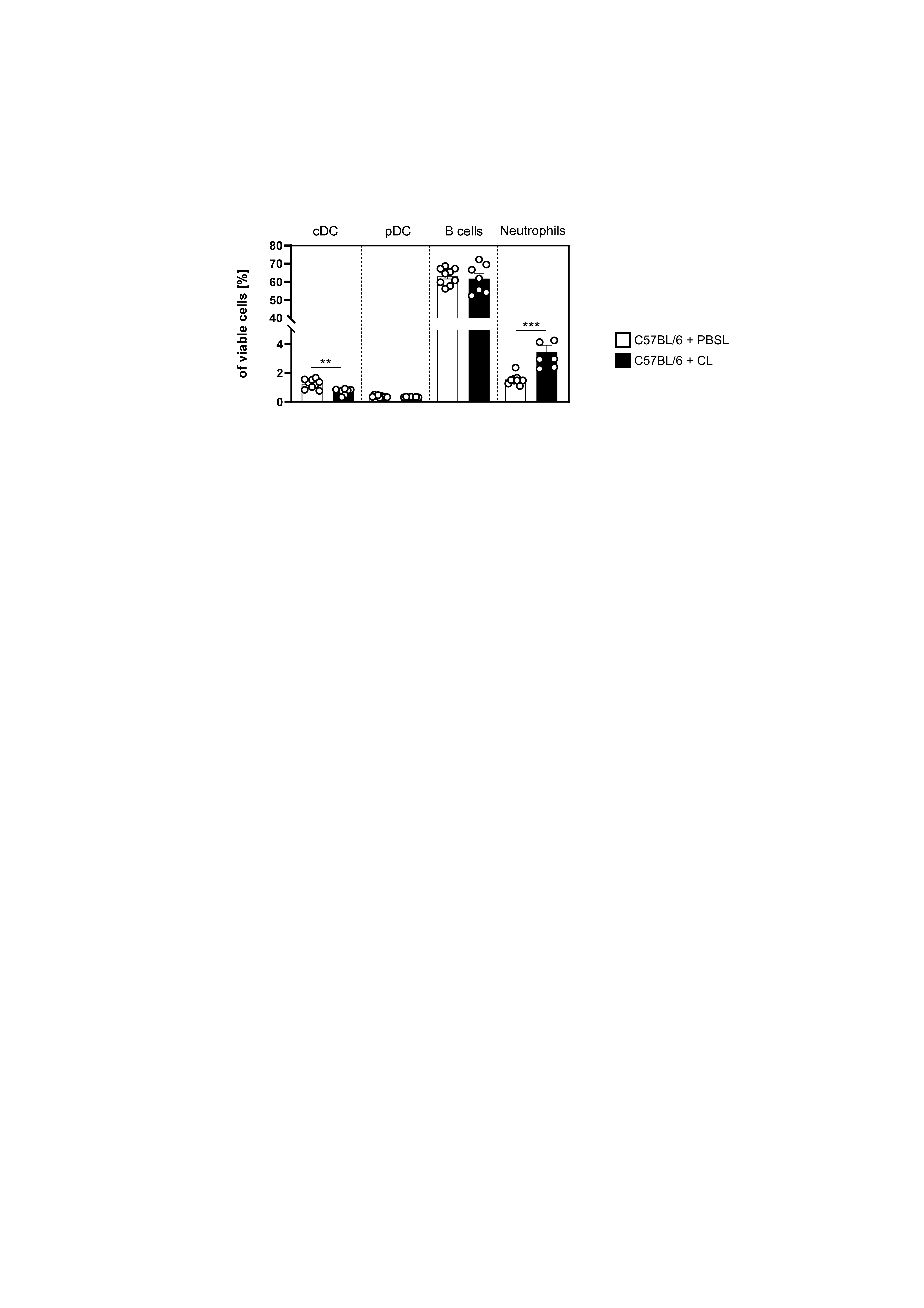


Supplemental Figure 4: Impact of CL administration on other immune cells

Frequencies of cDCs (CD11c^+^ CD317^-^), pDCs (CD11c^+^ CD317^+^), B cells (CD19^+^) and neutrophils (CD11b^+^ Ly6G^+^) of spleen from uninfected C57BL/6 mice treated with either PBSL or CL were analysed 10 days post liposome administration via flow cytometry. Results from 3 independent experiments with n=7-9 are presented as mean (± SEM). Data were analysed for statistical significance using unpaired Student’s t-test or Mann-Whitney test ***p* < 0.01, ****p* < 0.001.


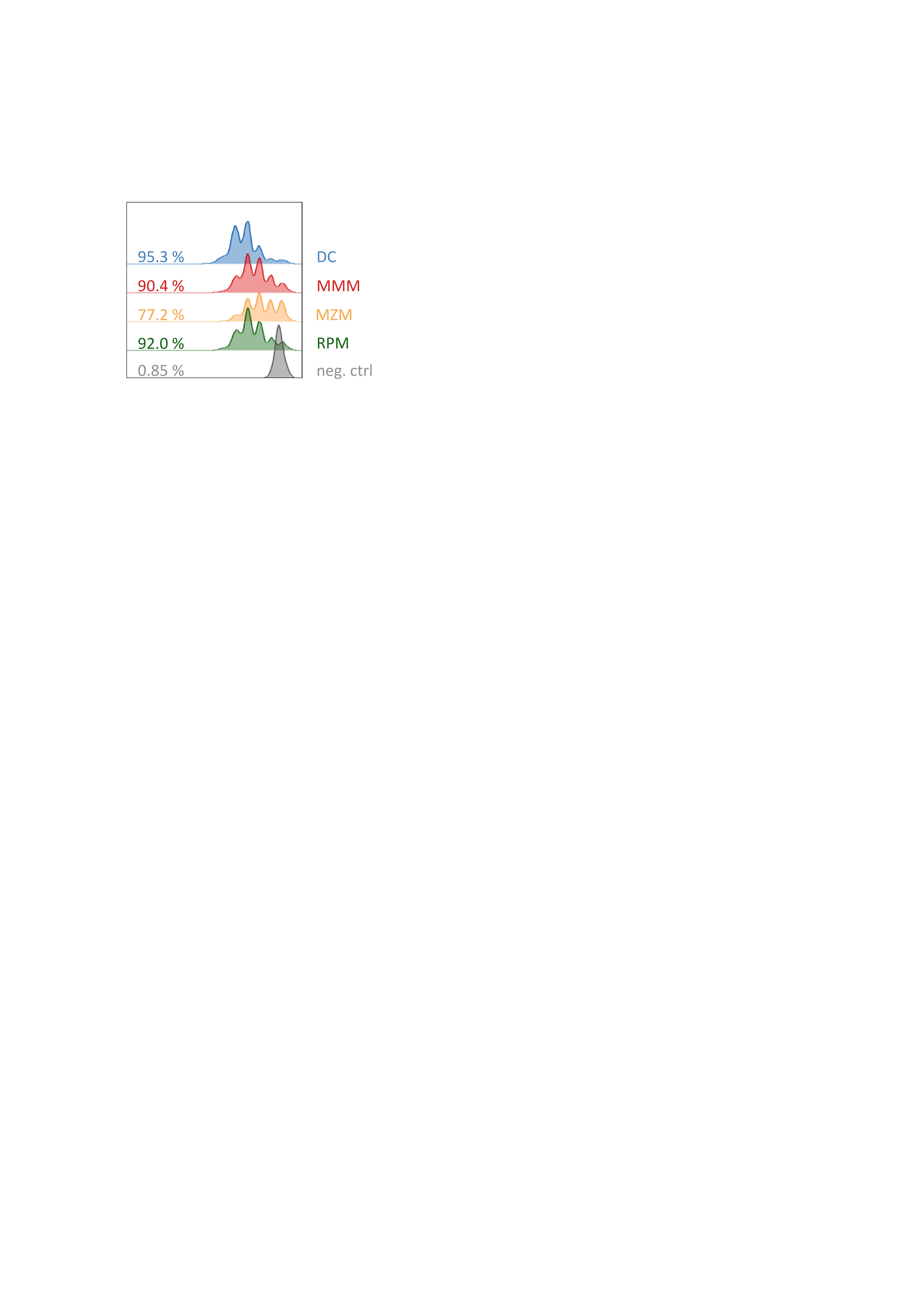


Supplemental Figure 5: Efficient induction of T cell proliferation macrophages *in vitro*

Proliferation of OVA-specific CD4^+^ T cells from OT-II mice stimulated with OVA-peptide loaded RPMΦ, MZMΦ, MMMΦ and DCs isolated from pooled spleens (n=1-3) of C57BL/6 mice or unstimulated CD4^+^ T cells (negative control).


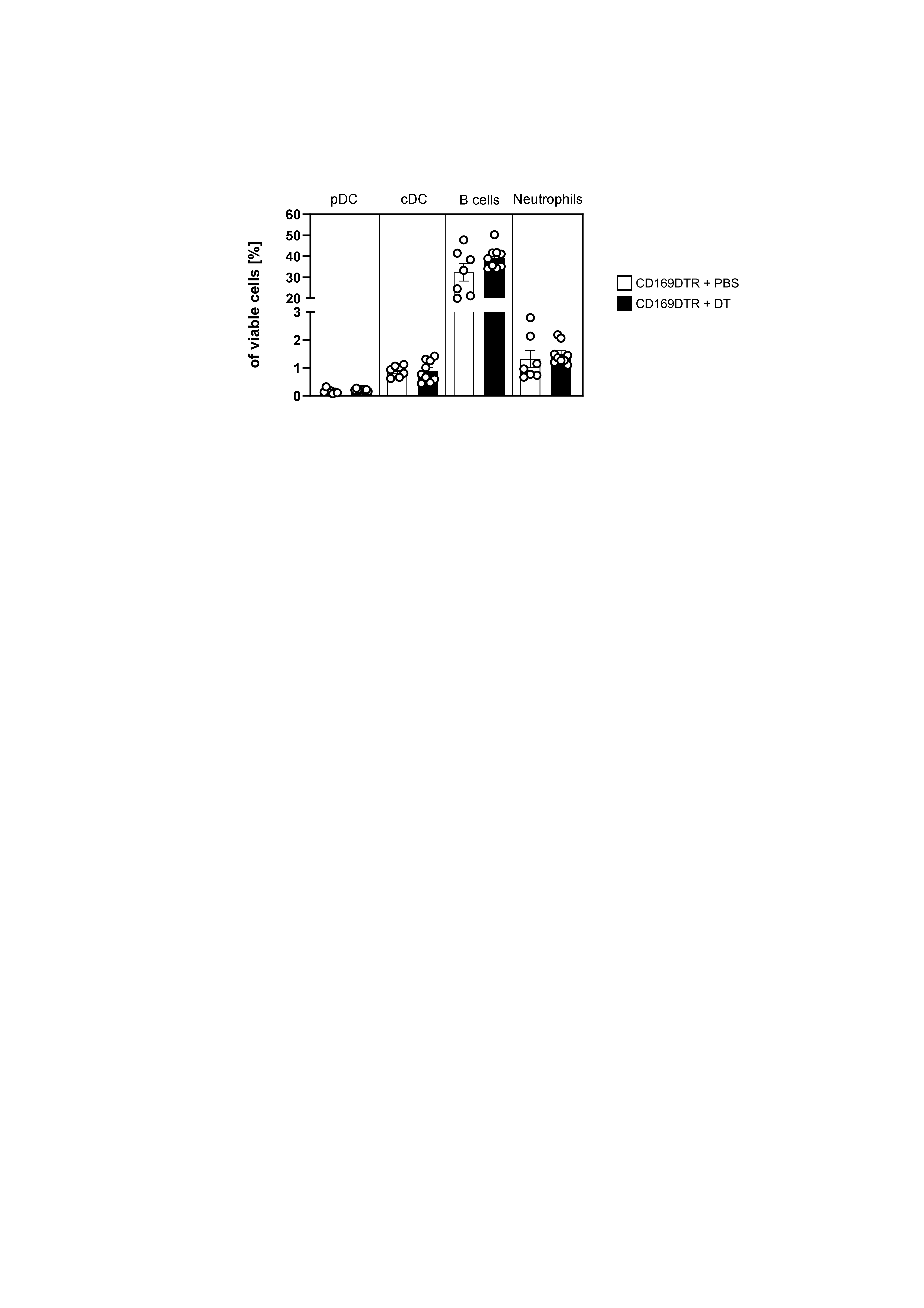


Supplemental Figure 6: Impact of DT administration in CD169DTR on other immune cells

Frequencies of cDCs (CD11c^+^ CD317^-^), pDCs (CD11c^+^ CD317^+^), B cells (CD19^+^) and neutrophils (CD11b^+^ Ly6G^+^) of spleen from uninfected CD169DTR mice treated with either PBS or DT were analyzed 8 days post PBS/DT administration via flow cytometry. Results from 3 independent experiments with n=7-9 are presented as mean (± SEM). Data were analysed for statistical significance using unpaired Student’s t-test or Mann-Whitney test.
