# Supplementary material for: Red pulp macrophages clear parasites, while marginal metallophilic and marginal zone macrophages support CD4+ T cell activation during Plasmodium yoelii infection
Source: Front Immunol. 2025 Jul 17;16:1607201. doi: 10.3389/fimmu.2025.1607201 (PMC12310687; doi:10.3389/fimmu.2025.1607201)
Supplement: Supplementary file 2 [file DataSheet2.pdf]

Supplementary Material

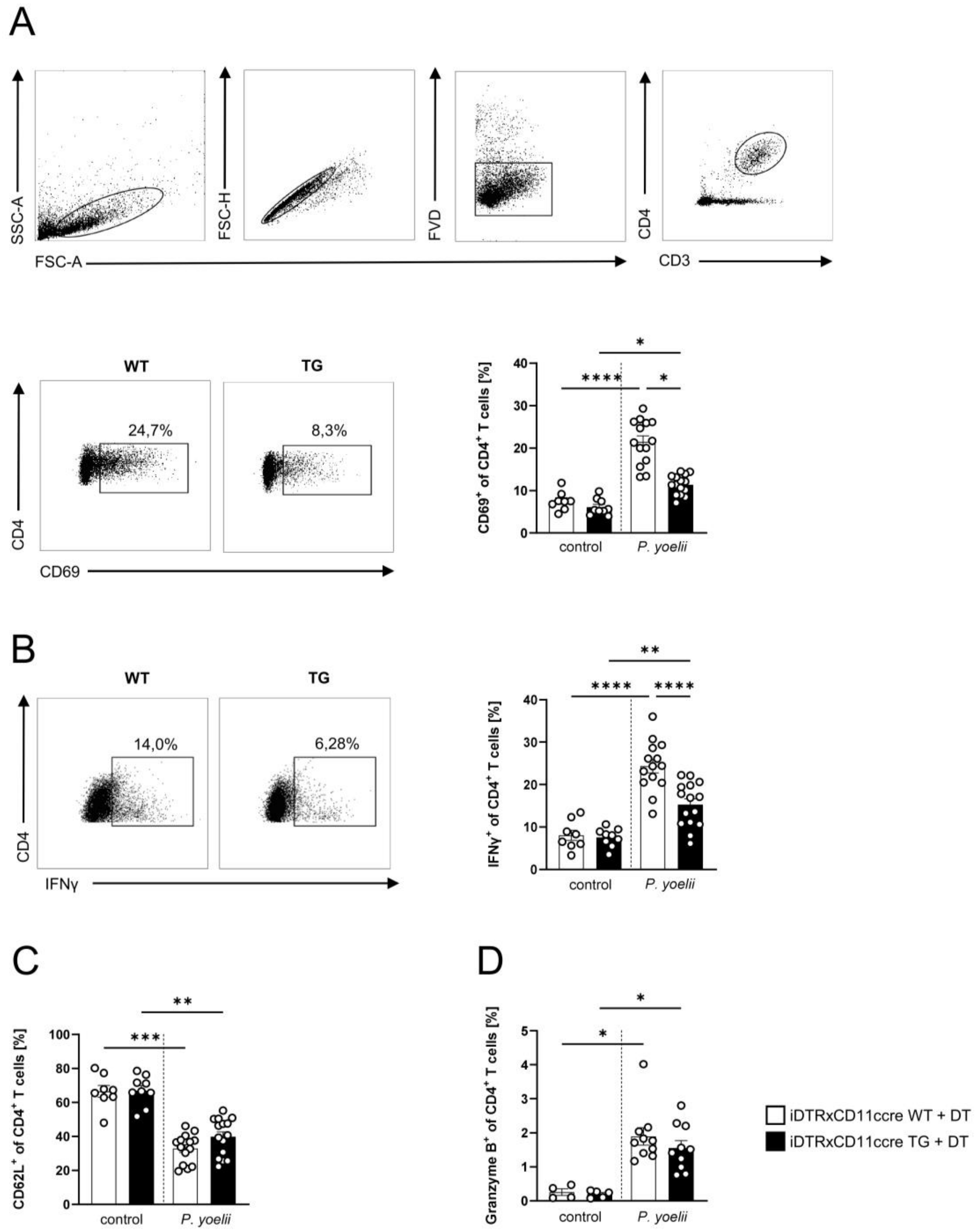

Supplemental Figure 1: DC-depleted mice show decreased CD4$^+$ T cell responses during *P. yoelii* infection

(**A**) Representative dot plots of isolated splenocytes and CD4$^+$ CD3$^+$ T cells that were analyzed via flow cytometry, as well as representative dot plot of CD69$^+$ CD4$^+$ T cells and summarized frequencies of CD69$^+$ CD4$^+$ T cells. (**B**) Representative dot plot of IFNγ$^+$ CD4$^+$ T cells and frequencies of IFNγ$^+$ CD4$^+$ T cells, (**C**) frequencies of CD62L$^+$ CD4$^+$ T cells and (**D**) granzyme B$^+$ CD4$^+$ T cells in spleen from uninfected (control) and *P. yoelii*-infected DT-treated iDTR x CD11ccre WT and TG mice that

were analyzed by flow cytometry 7 days p.i. Results from 3 independent experiments with n=6-15 are presented as mean (±SEM). Statistical analyses were performed using Kruskal-Wallis test or ordinary one-way ANOVA *$p < 0.05$, **$p < 0.01$, ***$p < 0.001$, ****$p < 0.0001$.

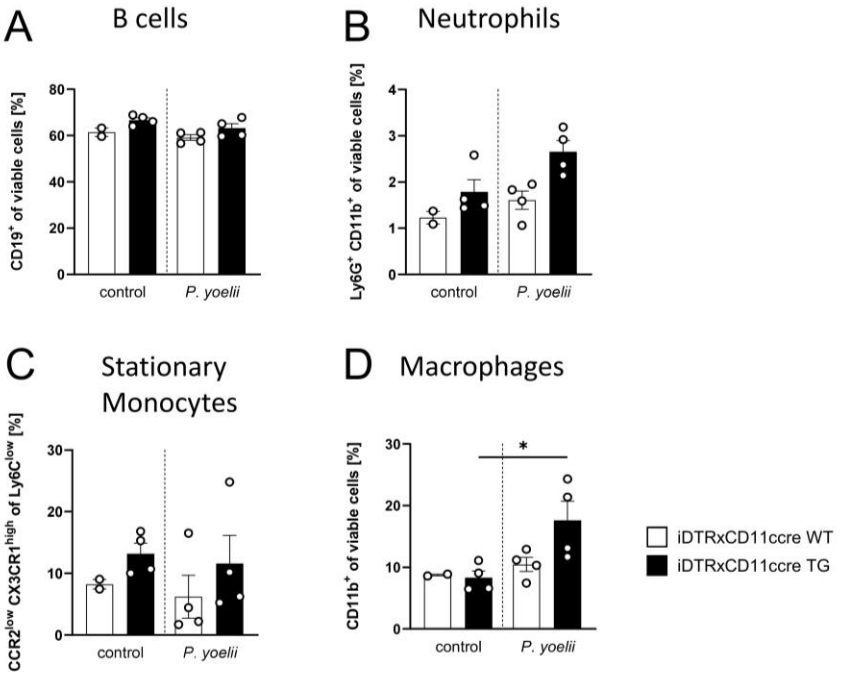

Supplemental Figure 2: DC depletion leads to an expansion of the splenic macrophage compartment

(**A**) Frequencies of $CD19^+$ B cells, (**B**) $Ly6G^+$ $CD11b^+$ neutrophils, (**C**) $CCR2^{low}$ $CX3CR1^{high}$ of $Ly6C^{low}$ cells presenting stationary monocytes and (**D**) $CD11b^+$ cells in spleen from uninfected (control) and *P. yoelii*-infected DT-treated iDTR x CD11ccre WT and TG mice 3 days p.i. Results from 1 experiment with n=2-4 are presented as mean (±SEM). Statistical analysis was performed using unpaired Kruskal-Wallis test *$p < 0.05$.

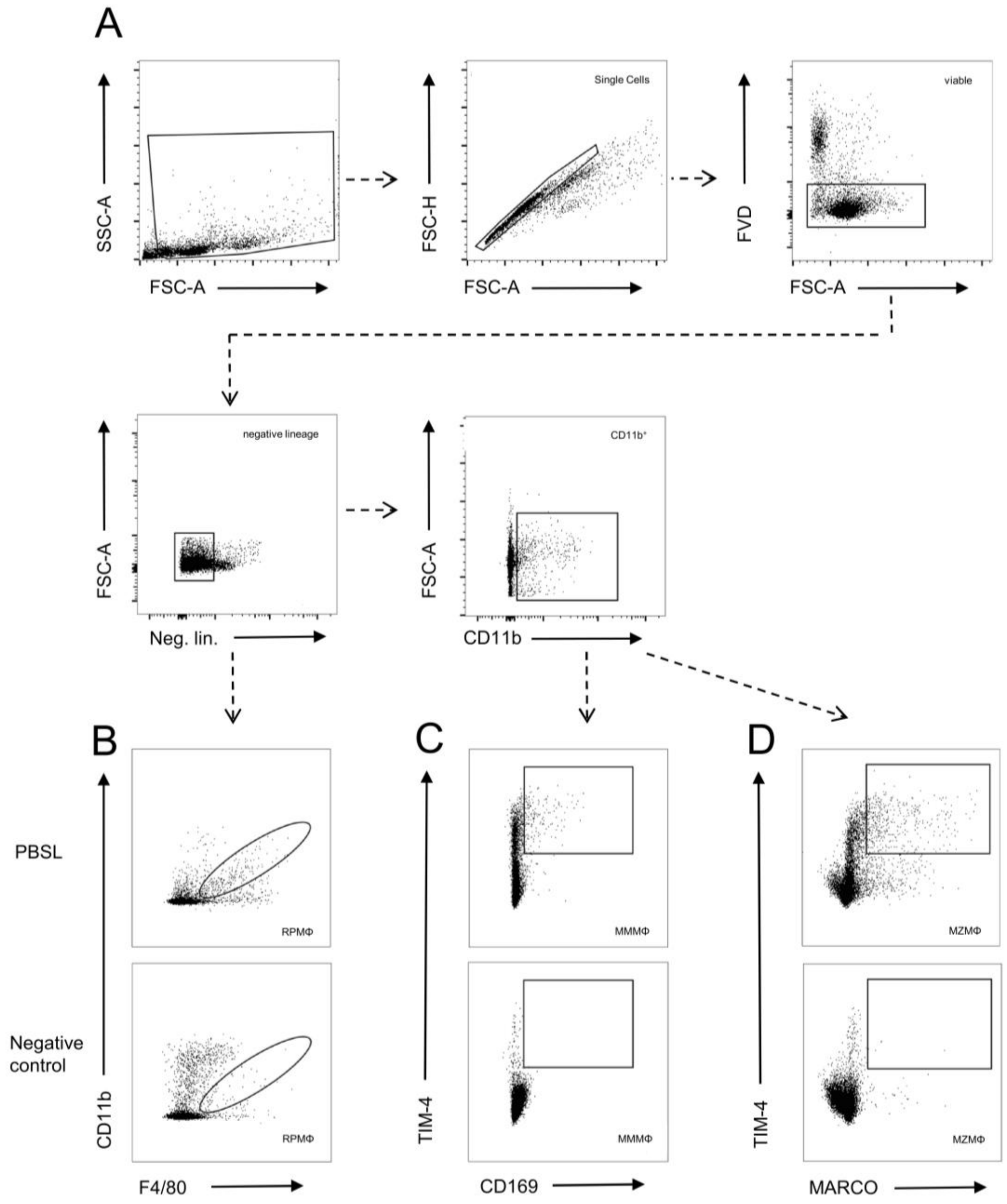

Supplemental Figure 3: Gating strategy of splenic macrophage subpopulations

(**A**) Representative flow cytometry gating strategy of splenic macrophage subpopulations of C57BL/6 WT mice treated with PBSL or CL as negative control from all cells to single and viable cells defined as FVD$^-$. (**B**) RPMΦ were defined as lineage negative (CD3$^-$ CD19$^-$ NK1.1$^-$ Ly6G$^-$) and F4/80$^+$ CD11b^{low} (**C**) MMMΦ were defined as lineage negative, CD11b$^+$ TIM-4$^+$ CD169$^+$ and (**D**) MZMΦ were defined as lineage negative, CD11b$^+$ TIM-4$^+$ MARCO$^+$.

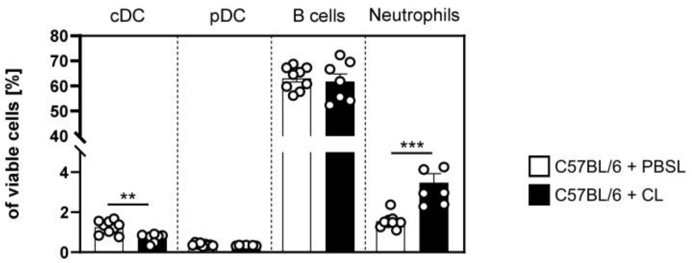

Supplemental Figure 4: Impact of CL administration on other immune cells

Frequencies of cDCs ($CD11c^+$ $CD317^-$), pDCs ($CD11c^+$ $CD317^+$), B cells ($CD19^+$) and neutrophils ($CD11b^+$ $Ly6G^+$) of spleen from uninfected C57BL/6 mice treated with either PBSL or CL were analysed 10 days post liposome administration via flow cytometry. Results from 3 independent experiments with n=7-9 are presented as mean (±SEM). Data were analysed for statistical significance using unpaired Student’s t-test or Mann-Whitney test $^{**}p < 0.01$, $^{***}p < 0.001$.

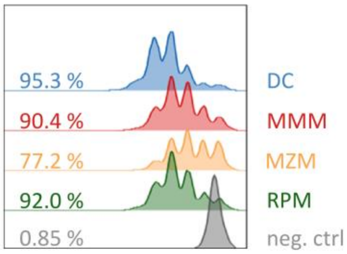

Supplemental Figure 5: Efficient induction of T cell proliferation macrophages *in vitro*

Proliferation of OVA-specific $CD4^+$ T cells from OT-II mice stimulated with OVA-peptide loaded RPMΦ, MZMΦ, MMMΦ and DCs isolated from pooled spleens (n=1-3) of C57BL/6 mice or unstimulated $CD4^+$ T cells (negative control).

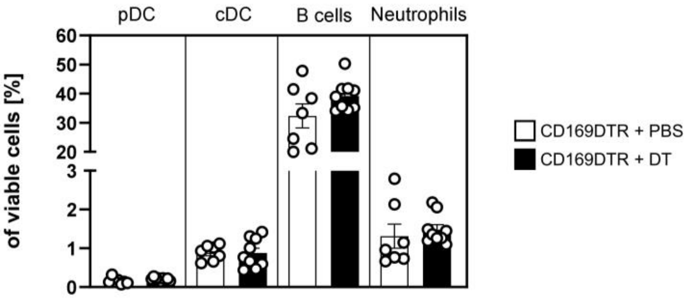

Supplemental Figure 6: Impact of DT administration in CD169DTR on other immune cells

Frequencies of cDCs (CD11c^{+} CD317^{-}), pDCs (CD11c^{+} CD317^{+}), B cells (CD19^{+}) and neutrophils (CD11b^{+} Ly6G^{+}) of spleen from uninfected CD169DTR mice treated with either PBS or DT were analyzed 8 days post PBS/DT administration via flow cytometry. Results from 3 independent experiments with n=7-9 are presented as mean (±SEM). Data were analysed for statistical significance using unpaired Student's t-test or Mann-Whitney test.
